# Treatment Outcomes and Trajectories of Change in Patients Attributing Their Eating Disorder Onset to Anti-obesity Messaging

**DOI:** 10.1097/PSY.0000000000000962

**Published:** 2021-06-19

**Authors:** Janell L. Mensinger, Shelbi A. Cox, Jennifer R. Henretty

**Affiliations:** From the M. Louise Fitzpatrick College of Nursing (Mensinger), Villanova University, Villanova, Pennsylvania; and Discovery Behavioral Health (Cox, Henretty), Center For Discovery, Los Alamitos, California.

**Keywords:** anti-obesity messaging, eating disorders, weight stigma, weight-inclusive care, higher levels of care, eating disorder treatment outcomes, **BMI** = body mass index, **CI** = confidence interval, **EDE-Q** = Eating Disorder Examination—Questionnaire, **ED(s)** = eating disorder(s), **EM** = estimated marginal, **EMM** = estimated marginal mean, **EMM Δ** = estimated marginal mean differences, **%TBW** = percent target body weight, **SE** = standard error, **TBW** = target body weight

## Abstract

**Objective:**

Given the increased prevalence of eating disorders (EDs) among individuals higher on the weight spectrum, we aimed to 1) report the prevalence of ED patients in higher levels of care (residential, partial hospitalization, and intensive outpatient) attributing the onset of their ED to anti-obesity messaging, 2) report the most commonly recollected sources of those messages, and 3) determine if those attributing the onset of their ED to anti-obesity messaging a) enter, b) exit, and c) respond to treatment differently from peers who did not.

**Methods:**

This retrospective cohort study used data from 2901 patients receiving ED treatment in higher levels of care at a US-based center between 2015 and 2018. Multilevel models examined differences in ED symptoms and trajectories of change over time. NVivo was used to analyze the patients’ comments about sources of messages.

**Results:**

Eighteen percent attributed their ED onset to anti-obesity messaging, 45% did not, and 37% were unsure. Of those providing comments, the most common sources included the following: educational curriculum/school context (45.9%), media/Internet (24.7%), health care (10.4%), family (9%), and peer bullying (3.7%). At admission, patients attributing their ED onset to anti-obesity messaging had more severe ED symptoms than those who did not (γ = 0.463, standard error [SE] = 0.086, *p* < .001) and those who were unsure (γ = 0.288, SE = 0.089, *p* < .001); no differences were evident at discharge (*p* > .483). During phase 2 of treatment, patients attributing their ED onset to anti-obesity messaging improved faster than those who did not (γ = 0.003, SE = 0.001, *p* = .008) and those who were unsure (γ = 0.003, SE = 0.001, *p* = .014).

**Conclusions:**

Anti-obesity messaging may put vulnerable individuals at risk for EDs. We recommend increasing weight bias training for school personnel and health care professionals. To reduce health disparities, we also suggest the promotion of weight-neutral health-enhancing self-care practices in media and public health campaigns, legislative policies, and health care overall.

## Abstract 2

Supplemental digital content is available in the text.

## INTRODUCTION

Eating disorders (EDs) are serious mental health conditions that cause significant health problems—even death—and early identification and treatment are crucial for best prognosis (1–3). Although estimated prevalence rates vary across the globe, one recent study of Australian adolescents showed 22.2% with a probable ED diagnosis from the *Diagnostic and Statistical Manual of Mental Disorders, Fifth Edition* (4), including the unspecified feeding and eating disorders (i.e., syndromes not fitting into the established criteria for specified disorders) (5). A recent systematic review of global prevalence rates demonstrated a doubling of ED point prevalence since the early 2000s (from 3.5% to 7.8%, across all ages) (6). Furthermore, several epidemiological studies show alarming increases in disordered eating among people with body mass index (BMI) greater than 30 kg/m^2^ (7–9). Although these increases may be in part due to new attention on the historically underrecognized restrictive EDs in higher-weight people (10,11), some researchers speculate that these increases are a consequence of widespread public health campaigns around the dangers and problems related to high BMI (12–15).

Hunger and Tomiyama (16) recently used modified labeling theory to investigate the potential harm in messages about weight. The approach posits that terms used to describe an individual will contribute to their identity development and influence future behavior. More specifically, modified labeling theory suggests that the damaging and stigmatizing effects of a label occur because, no matter how well intentioned the act, identifying someone as a member of any socially devalued group often leads to internalization of negative societal stereotypes about the group (17). Consistent with this theory, their study demonstrated that classifying an adolescent girl as “too fat” was associated with greater disordered behaviors and cognitions 5 years later, and this effect was especially pronounced when the source of the labeling was a family member (16). Indeed, family members have been cited as the most common source of weight stigma, followed by doctors and then classmates (18).

Although identification of a child’s elevated weight status is traditionally considered an important method of obesity prevention, research has shown insufficient data to recommend for or against BMI surveillance for youth (19–21). In fact, a recent large cluster randomized clinical trial of 28,641 California students in grades 3 through 8 found no changes in BMI *z*-scores among higher-weight students (>85th percentile) in schools implementing the BMI screening protocol (22). Moreover, compared with the control group students (i.e., schools with no BMI surveillance protocol), body dissatisfaction and peer weight talk increased significantly more among students in the schools that were assigned the BMI screening (22).

As modified labeling theory implies, other studies also suggest weight surveillance may come with unintended consequences, especially if not implemented with the Center for Disease Control’s recommended safeguards, which few schools do (23–25). Using an econometric model allowing for causal inference, a study of the New York City school system showed girls given the “overweight” label in their BMI report card had significantly greater BMI gains the following year compared with the control group girls not labeled (26). Although the negative effect was small overall, it became more pronounced for older girls (i.e., those in their junior year) and for those never previously labeled “overweight.” There was no labeling effect for boys (26).

In related research, adolescent girls participating in the National Heart Lung and Blood Institute’s Growth and Health Study who reported being identified as “too fat” also had significantly higher odds of an “obese” BMI 10 years later, even after adjusting for baseline BMI, race, income, and parental education (27). Similarly, adolescents with BMIs in the “overweight” and “obese” range from the National Longitudinal Study of Adolescent to Adult Health wave II cohort were followed up to determine how their perceived weight influenced future weight change (28). Those who inaccurately described themselves as having a “normal” weight had significantly lower BMI gains after 13 years, even after adjusting for baseline BMI (28). In a connected line of work, Schvey and colleagues (29) showed that perceived pressures to be thin were associated with insulin resistance in adolescent boys and girls, and the relationships were maintained when adjusting for body composition (i.e., percent lean mass and kilograms of fat mass).

Comparable associations hold in adult samples. Perceiving oneself as “overweight,” whether the perception was medically accurate or not, predicted future weight gain in more than 14,000 adults from the United States and United Kingdom, which, again, held true after baseline BMI adjustment (30). In addition, a study of 3582 adults in the United States implied that messages warning individuals of their “weight problems” do not predict health improvements in the long run (31). To the contrary, researchers found that perceptions of being “overweight,” irrespective of actual BMI at baseline, were associated with worse long-term physiological dysregulation and poorer subjective health ratings after a 7-year period compared with those without such perceptions (31). In a nationally representative sample of 6157 adults, those reporting weight-based discrimination were also more likely to have increased their weight by follow-up, adjusting for baseline BMI, age, sex, ethnicity, and education (32). In sum, through multiple samples and methodologies, these studies show the ineffectiveness and potential harm of labeling strategies for higher-weight individuals.

Multiple studies also experimentally demonstrated the dangers of messages about food and body in laboratory environments. For instance, after exposure to dieting products and slender models, self-identified restrained eaters further restricted food intake (33). Likewise, body dissatisfaction increased after viewing “thin-and-beautiful” media images (34). Moreover, Puhl et al. (35) showed that stigmatizing ads induced less self-efficacy for change in health-related behaviors than did neutral messages in a large randomized trial of the American public’s (*n* = 1085) reactions to weight-related health campaigns. This finding supports earlier research suggesting weight stigma is associated with exercise avoidance, as well as a whole host of negative sequelae: physiological, psychological, and behavioral (36,37). It also sheds light on the problem that, per a recent review, 44% of obesity-related public health campaigns included a stigmatizing strategy, even though research shows people evaluate messages as more helpful and motivating when they are *not* stigmatizing—or weight based at all—but rather focus solely on healthy behaviors (15,38).

This research underpins cross-sectional evidence supporting an expanded tripartite influence model of ED risk whereby family, peer, partner, and media pressures about weight are associated with disordered eating through the internalization of the thin ideal, and over a decade of studies showing that internalized and experienced weight stigma are related to disordered eating (39–43).

Finally, although it is well known that EDs have a multifactorial etiology involving an interplay of psychosocial, environmental, and genetic risk (44), dieting behaviors—especially extreme weight control practices (e.g., fasting, diet pills, laxative use)—have long been conceptualized as fundamental precursors for EDs (45). Although the relationship is nuanced (i.e., not all diets lead to EDs) (46), this link has been established through experimental frameworks, as shown in restraint theory (47,48), and through longitudinal research on the relationship between early weight-control behaviors and the later development of disordered eating and/or diagnosed clinical EDs (49,50).

Thus, a large number and wide variety of studies raise critical concerns surrounding the ethics of using weight-related public health messaging (51). Considering weight loss interventions do not consistently lead to the oft-presumed health benefits (52–57), particularly in the long term (58), and given that dieting is also associated with weight cycling, which escalates both morbidity and mortality (59–63), we must exercise caution with the use of anti-obesity public health messaging.

The present research seeks to underscore the need for attention to the potential harm of weight-based public health interventions and the consequential trickle-down effects of these interventions, from government-mandated policy change of the public school health curriculum to advertising campaigns and even one-on-one individualized messaging (e.g., from well-meaning health care providers and family members). Health researchers have long warned of iatrogenic effects of public health interventions, particularly toward prevention of “obesity” in children (23–25,64). Although Bonell and colleagues (65) have theorized about methods for uncovering mechanisms of harm in public health interventions, such theory is rarely evaluated. Our goal is to investigate one component of this matter in a large sample of patients in treatment for an ED by documenting reports of anti-obesity messages as the factor prompting the onset of their ED.

Despite the widespread knowledge that sociocultural pressures toward thinner body norms are among the most robust risk factors of EDs (44), to our knowledge, no previous studies have examined how often patients entering treatment for an ED attribute the onset of their disorder to anti-obesity messaging, and whether those who do are any more or less symptomatic at treatment admission and discharge than their peers whose EDs were not triggered by anti-obesity messaging. Therefore, the specific aims of this study are to 1) report the prevalence of ED patients in higher levels of care (i.e., residential, partial hospital program, and intensive outpatient treatment) attributing the onset of their ED to anti-obesity messaging, 2) report the most commonly recollected sources of those messages, and 3) determine if patients attributing the onset of their ED to anti-obesity messaging a) enter, and b) exit treatment with more or less severe symptoms, and c) respond to treatment at a faster or slower rate than peers who did not attribute the onset of their ED to anti-obesity messages.

## METHODS

### Participants and Setting

The study was a retrospective cohort design of 2901 patients receiving treatment for a diagnosed ED at a US-based ED specialty center. ED diagnosis was given by the referring clinician and confirmed by the intake clinician upon admission to treatment. The American Psychiatric Association’s Practice Guidelines for the Treatment of Patients with EDs (66), along with the medical necessity criteria defined by the third-party insurance payors, were used for determination of eligibility into the treatment settings. Diagnoses ranged across the full spectrum of *Diagnostic and Statistical Manual of Mental Disorders, Fifth Edition* EDs: 1) anorexia nervosa–restricting type; 2) anorexia nervosa–binge/purging type; 3) bulimia nervosa; and 4) binge eating disorder; 5) avoidant/restrictive food intake disorder; and 6) other specified feeding and eating disorder, a category that includes a) purging disorder, b) atypical anorexia nervosa (i.e., when all features of anorexia nervosa are met except that despite weight loss, the individual’s weight remains within or above a “normal” range), c) night eating syndrome, d) low-frequency or short-duration bulimia nervosa, and e) low-frequency or short-duration binge eating disorder.

**FIGURE 1:**
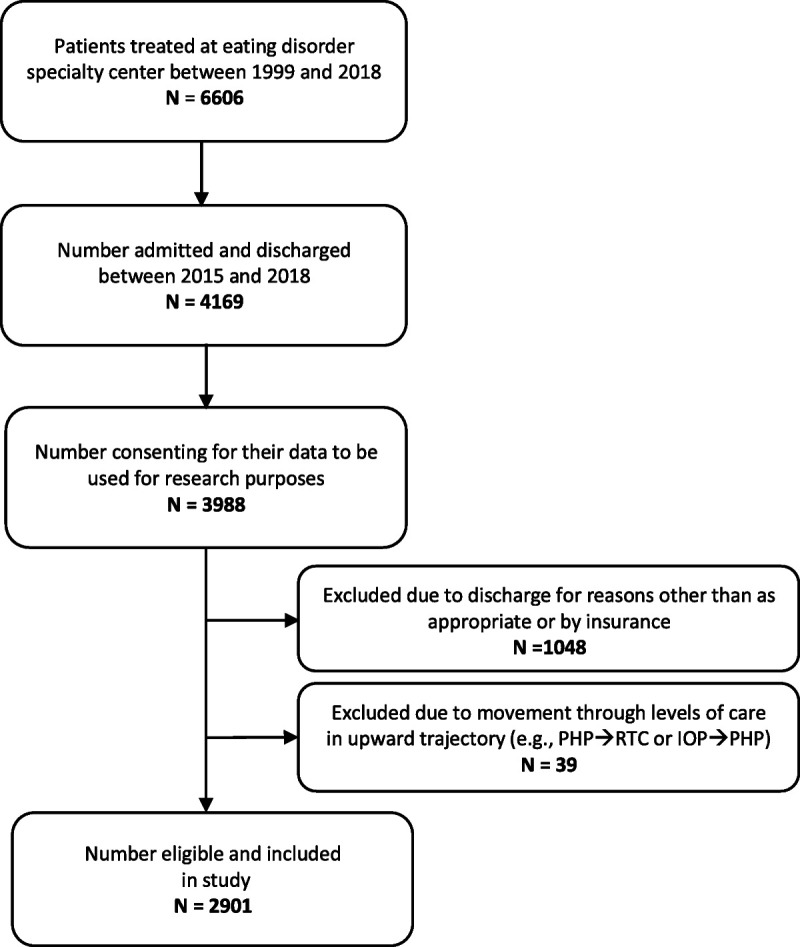
STROBE flow diagram of participants included in the study sample. STROBE = Strengthening the Reporting of Observational Studies in Epidemiology; RTC = residential treatment center; PHP = partial hospital program; IOP = intensive outpatient program.

We extracted deidentified data from the center’s electronic medical records based on the inclusion criteria of the first and most complete, contiguous treatment involving a downward progression in intensity of care (i.e., residential treatment ➔ partial hospital ➔ intensive outpatient) whereby patients were discharged to the next lower level of care as appropriate (and/or determined by insurance), between the years of 2015 and 2018. These criteria ensured there were no repeated cases in the data set. To best assure available data for longitudinal analysis, we excluded a patient’s first episode if a later episode was more intensive and involved moving through multiple levels of care. All patients included in the database had consented to have their information available for future research studies (see Figure 1 for the STROBE flow diagram). The Drexel University Institutional Review Board reviewed the protocol and provided a letter of determination that the research was considered “exempt.”

Most of the sample (*n* = 2094; 72%) was admitted to residential treatment (24-hour care); 478 (16%) initiated treatment in a partial hospital program (typically 5–6 d/wk, 8 h/d), and 329 (11%) began in intensive outpatient (typically 3–4 d/wk, 4 h/d). The final person-period data set included 7663 observations: 2061 had one level of care consisting of admission and discharge (79% of which were residential), 565 people had two levels of care consisting of two admissions and two step-downs (65% of which were partial hospitalization to intensive outpatient), and 275 had all levels of care encompassing three admissions and three step-downs.

### Procedures for Variable Measurement

Variables drawn from the electronic medical records included age, gender identity, race/ethnicity, ED diagnosis, and percent target body weight (%TBW) at admission. Percent TBW was determined by the Hamwi method for adults (http://www.scymed.com/en/smnxpn/pndfc237.htm) and the following formula for adolescents (<18 years): (50th percentile BMI × height in inches^2^) × (2.2). We collected %TBW as opposed to BMI, given the large proportion of adolescent patients in the sample; BMI is a less reliable measurement for capturing weight-to-height ratio in adolescence. The correlation between BMI and %TBW was 0.99 in the current data set. As part of the standard, structured biopsychosocial intake completed by a licensed therapist (or under the supervision of one), patients were asked about the months since the onset of their ED; prior ED treatment; and history of bullying, sexual abuse, and other forms of trauma (e.g., physical, emotional, and verbal abuse, which were combined to form a single “other trauma” variable).

The study’s primary exposure variable—whether the ED onset was attributed to anti-obesity messaging—was charted by the intake clinician. Clinicians were trained to perform all questioning and response coding for the structured biopsychosocial intake in a standardized format. After being asked how old they were when their ED started, patients were next asked “Can you remember if there was something that happened that led to the start of the ED.” If the response involved an anti-obesity messaging trigger, the exposure was coded as “yes,” and the clinician probed further by asking “What was the primary source of those messages?” Patients who were unsure of what triggered their ED received an “unsure” for the anti-obesity messaging variable. Patients naming other factors leading to their ED received a “no.”

The Eating Disorder Examination—Questionnaire (EDE-Q) was completed at admission and discharge from each level of care as a measure of treatment success (67). The EDE-Q is a well-validated, self-report measure of ED severity that is among the most widely adopted transdiagnostic tools to assess ED treatment outcomes (68). It has established normative data for clinical populations, and it has been shown to be responsive to treatment effects (69–71). The EDE-Q asks patients to evaluate the extent to which they have experienced cognitive and behavioral components of EDs over the past 28 days on a scale from 0 (no days) to 6 (every day). Global scores were calculated as the study outcome variable; higher scores indicate greater symptom severity.

The maximum number of timepoints per participant was six, an admission and discharge for each level of care. About three-quarters of the sample were initially admitted into residential treatment. However, given that some patients began treatment in partial hospital or intensive outpatient, reducing the number of timepoints available for them, we created an indicator variable for level of care admission to allow adjustment for this in all models. The time variable used for the models presented is the exact day of assessment, counting from initial admission as day 0 to each subsequent discharge and/or step-down admission assessment.

### Data Analysis

Data were imported into SPSS (version 25) and cleaned and analyzed using SPSS and SAS (version 9.4). Exploratory analyses included examining variable distributions with stem-and-leaf and box-and-whisker plots to detect outliers and distribution problems. Number of months since ED onset was log transformed to normalize right skewness. Model diagnostics were performed by plotting the predicted against observed values and examining multivariate normality with histograms of the residuals. We also plotted the intercepts and slopes to establish that multivariate normality was met for the random coefficients portion of the models. Model-based estimates showed excellent fit to the observed data. One-way analyses of variance, χ^2^ tests, and Kruskal-Wallis analyses of variance were applied, as appropriate, to show whether there were differences on the covariates between patients attributing their ED onset to anti-obesity messaging and those who did not or were unsure. Bivariate Pearson correlations between the continuous covariates were examined for potential multicollinearity problems. Given the high correlation between months since ED onset and age (*r* = 0.84), we created a dichotomized age variable (<19 years).

To answer aim 1, frequencies and proportions were calculated to determine the prevalence of attributing anti-obesity messages to ED onset. For aim 2, we used thematic content analysis (72) initiated by a word frequency query in NVivo to identify content themes on the descriptive responses of the source of the anti-obesity messages. Subsequent text search queries were run on the most frequently occurring words identified in the responses. The process was completed by combining themes into categories (e.g., health class, physical education, teacher comments, and cooking class were combined into a single broad category to capture the educational curriculum and school environment).

Aim 3 was answered using multilevel mixed-effects models, which examined subject-specific differences in symptom severity a) at initial admission, b) at discharge, and c) trajectories of symptom change through treatment. To determine the best-fitting function(s) for time (aim 3c), empirical Bayes plots were fit to a random set of 50 observations to detect underlying trends for the trajectory of symptom change. Combining this information as an initial guide indicating nonlinear change, in accordance with practices consistent with Singer and Willet’s (73) framework for applied longitudinal analysis, we fit a series of unconditional growth models using various nonlinear functions of time to best represent trajectories of change through treatment. The final unconditional growth model (plus adjustments for initiating level of care; Supplemental Digital Content 1, http://links.lww.com/PSYMED/A753, Model 1) was chosen using the likelihood ratio test and examining Akaike criterion for the best model fit.

The best-fitting model included a random intercept function, which captures person-specific initial status of the EDE-Q at intake, and two random slope functions (a reciprocal function of time and linear time function) to represent the person-specific modeling of initial acceleration of change in symptoms at the beginning of treatment (reciprocal function) and a flatter change (linear function) captured during the second half of treatment. Subsequent models were fit to answer aim 3 without adjustments (model 2) and with confounder adjustments (models 3). The confounder-adjusted model shows the primary exposure variable of interest (anti-obesity messaging), plus the following *a priori* covariates: age; gender identity; race/ethnicity; ED diagnosis; prior treatment; %TBW at admission; and history of sexual abuse, bullying, and other trauma—all chosen based on theory and past research in the ED field about the importance of severity markers (e.g., length of illness, prior treatment), demographics (e.g., age, race, gender identity), and abuse-related risk factor differences (70,74–77). To preserve degrees of freedom and maintain a parsimonious model, covariates not reaching *p* < .10 were dropped; all covariates remained on at least one of the model levels (i.e., initial status, slope during phase 1, or slope during phase 2). Models are presented per Singer and Willet (73) style in Supplemental Digital Content 1, http://links.lww.com/PSYMED/A753.

Because multilevel models use maximum likelihood estimation, which keeps participants in the analysis as long as they have at least one observed outcome (all 2901 participants had a minimum of two timepoints), only missing values on covariates impacted the models. Given this problem was minimal (see available *n* values in Table 1), we were able to use more than 95% of the full sample. A sensitivity analysis on the complete sample (minus the 16 cases missing on the anti-obesity messaging trigger, 0.55%) for Model 2 was conducted, which did not include covariates, to confirm robustness of the results regarding the primary exposure variable. Multiple sensitivity analyses with subsets of the data were also performed.

**TABLE 1:** Baseline Participant Characteristics

Variable	Attributed ED Onset to Anti-obesity Messaging				
	Yes (*n* = 522)	No (*n* = 1310)	Unsure (*n* = 1053)	Total (*n* = 2885)	*p^a^*
	*n* (Row %)		*n* (Column %)		
Entry level of care (*n* = 2885)					.14
Residential treatment center	397 (19.0)	920 (44.1)	768 (36.8)	2085 (72.3)	
Partial hospital program	72 (15.1)	231 (48.5)	173 (36.3)	476 (16.5)	
Intensive outpatient program	53 (16.4)	159 (49.1)	112 (34.6)	324 (11.2)	
ED diagnosis (*n* = 2883)					.028
Anorexia nervosa—restricting	219 (17.7)	572 (46.1)	449 (36.2)	1240 (43.0)	
Anorexia nervosa–binge/purging	87 (17.6)	237 (47.9)	171 (34.5)	495 (17.2)	
Bulimia nervosa	101 (19.0)	220 (41.4)	211 (39.7)	532 (18.5)	
Binge ED	34 (17.3)	101 (51.3)	62 (31.5)	197 (6.8)	
ARFID	7 (8.4)	47 (56.6)	29 (34.9)	83 (2.9)	
OSFED	73 (21.7)	133 (39.6)	130 (38.7)	336 (11.7)	
Prior treatment (*n* = 2884)					.22
Yes	428 (18.7)	1026 (44.9)	833 (36.4)	2287 (79.3)	
No	94 (15.7)	283 (47.4)	220 (36.9)	597 (20.7)	
Race/ethnicity (*n* = 2786)					.048
White non-Hispanic	379 (17.5)	1013 (46.8)	773 (35.7)	2165 (77.7)	
Latinx	72 (21.3)	134 (39.6)	132 (39.1)	338 (12.1)	
Asian/Pacific Islander	14 (11.3)	62 (50.0)	48 (38.7)	124 (4.45)	
Black	13 (26.5)	20 (40.8)	16 (32.7)	49 (1.76)	
Multiracial	25 (26.3)	34 (35.8)	36 (37.9)	95 (3.41)	
American Indian/Native Alaskan	3 (20.0)	7 (46.7)	5 (33.3)	15 (0.54)	
Gender identity (*n* = 2885)					.047
Female	502 (18.5)	1220 (45.1)	986 (36.4)	2708 (93.9)	
Male	20 (11.3)	90 (50.8)	67 (37.9)	177 (6.1)	
Age group (*n* = 2885), y					.14
≥19	205 (39.3)	571 (43.6)	425 (40.4)	1632 (58)	
≤18	317 (60.7)	739 (56.4)	1053 (59.6)	1181 (42)	
History of sexual abuse (*n* = 2881)					.84
Yes	146 (17.5)	381 (45.6)	309 (37.0)	836 (29.0)	
No	376 (18.4)	926 (45.3)	743 (36.3)	2045 (71.0)	
History of other trauma (*n* = 2885)					.081
Yes	278 (19.7)	632 (44.8)	500 (35.5)	1410 (48.9)	
No	244 (16.5)	678 (46.0)	553 (37.5)	1475 (51.1)	
History of being bullied (*n* = 2879)					<.001
Yes	306 (21.6)	589 (41.5)	524 (36.9)	1419 (49.3)	
No	216 (14.8)	718 (49.2)	526 (36.0)	1460 (50.7)	
	Median (IQR)				
Months since ED onset (*n* = 2870)	36.0 (12–96)	37.5 (14–108)	36.0 (13–96)	36.0 (13–108)	.781
Age (*n* = 2885), y	17.0 (15–21)	17.0 (15–24)	17.0 (15–22)	17.0 (15–23)	.239
	Mean (SD)			
% Target body weight (*n* = 2885)	109.4 (35.7)	104.0 (32.6)	106.9 (37.3)	106.0 (35.0)	.007
EDE-Q score (*n* = 2567)	3.9 (1.5)	3.4 (1.7)	3.6 (1.7)	3.6 (1.7)	<.001

ED = eating disorder; ARFID = avoidant/restrictive food intake disorder; OSFED = other specified feeding and eating disorder; ED = eating disorder; IQR = interquartile range; EDE-Q = Eating Disorder Examination—Questionnaire.

*^a^ p* Values are for the overall omnibus test for χ^2^ on categorical variables, the omnibus *F* statistic for one-way analysis of variance on variables reporting means, and Kruskal-Wallis for the variables reporting medians.

## RESULTS

Table 1 displays the patient characteristics broken down by whether they attributed their ED onset to anti-obesity messaging (yes, no, unsure). The sample had a mean (SD) age of 21.7 (7.32) years (range, 9–83 years). Because of the age distribution’s extreme right skew, in Table 1, we present the median (interquartile range) of 17 (15–23) years. The large majority of the sample was female identifying (94%; *n* = 2708) and the remaining 6% (*n* = 177) was male identifying; 0.5% (*n* = 16) had no response to the gender identity question. The most frequent ED diagnosis was anorexia nervosa–restricting type (43%; *n* = 1240), 19% (*n* = 532) had a diagnosis of bulimia nervosa, 17% (*n* = 495) were diagnosed with anorexia nervosa–binge/purging type, 12% (*n* = 336) had a diagnosis of other specified feeding and eating disorders, 7% (*n* = 197) were diagnosed with binge eating disorder, and 3% (*n* = 83) were diagnosed with avoidant/restrictive food intake disorder. Most patients (78%; *n* = 2165) identified as White non-Hispanic, 12% (*n* = 338) as Latinx, 4% (*n* = 124) as Asian or Pacific Islander, 2% (*n* = 49) as African American or Black, 1% (*n* = 15) as American Indian or Native Alaskan, and 3% (*n* = 95) as multiple races/ethnicities. No racial/ethnic identity information was provided for 115 patients.

Of the 2901 patients, 522 (18%) attributed their ED onset to anti-obesity messaging, 1053 (37%) were unsure if anti-obesity messaging precipitated their ED, and the remaining 1310 (45%) attributed it to other factors. Content analysis of the descriptive data showed that the most commonly recollected source of anti-obesity messages in this patient sample was related to the theme of education curriculum and school environment (45.9% of the 490 patients who named sources of anti-obesity messages). Sources of messaging from the educational curriculum theme included reference to classes and teachers in general, as well as health, nutrition, cooking, and, physical education classes more specifically. The second most common source of messaging came from the Internet/social media and general media (24.7%). Other themes included messaging from health care providers (10.4%), family members (9%), and peer bullying (3.7%). Not all sources (e.g., church) fell under identified themes.

**FIGURE 2:**
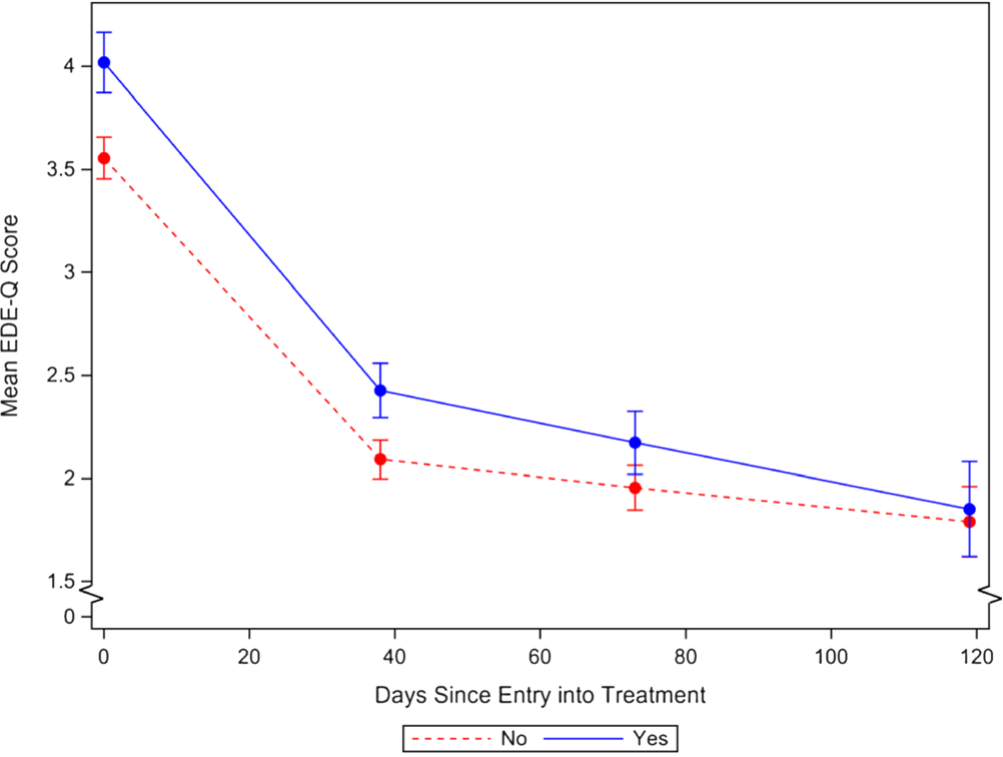
Estimated global Eating Disorder Examination—Questionnaire (EDE-Q) score trajectories of change. Dashed line (no) represents patients not attributing eating disorder onset to anti-obesity messaging. Solid line (yes) represents patients attributing eating disorder onset to anti-obesity messaging. Error bars represent 95% confidence intervals. First point represents predicted intake global EDE-Q score for patients entering residential treatment; second point represents predicted residential discharge score after a median length of stay of 38 days; third point represents predicted partial hospital discharge score after a median length of stay of 73 days; fourth point represents predicted intensive outpatient program discharge score after a median length of stay of 119 days. Color image is available online only at www.psychosomaticmedicine.org.

Patients who attributed their ED onset to anti-obesity messaging had significantly higher EDE-Q scores at admission than those who did not and those who were unsure, with estimated marginal mean differences (EMM Δ) of 0.463 (95% confidence interval [CI] = 0.295 to 0.631) and 0.288 (95% CI = 0.114 to 0.462), respectively. By final discharge, however, these differences were no longer evident for the “yes” versus “no” groups (EMM Δ = 0.062, 95% CI = −0.197 to 0.321) or the “yes” versus “unsure” groups (EMM Δ = −0.096, 95% CI = −0.366 to 0.173). Tests of differences in rates of change over the first phase of treatment (captured in the reciprocal time trend) showed that patients attributing their ED onset to the anti-obesity messaging were no different from their peers who did not (γ = −0.008, standard error [SE] = 0.124, *p* = .950) or their peers who were unsure (γ = 0.062, SE = 0.128, *p* = .625), meaning when patients with the anti-obesity message trigger were discharged from the *initial* phase of treatment, they remained more symptomatic than their peers without the trigger. However, during the latter phase of care (captured in the linear time trend), patients attributing their ED onset to anti-obesity messaging improved significantly faster than those who did not (γ = 0.003, SE = 0.001, *p* = .008) and those who were unsure (γ = 0.003, SE = 0.001, *p* = .014), showing final discharge at equal levels of symptom severity. After adjusting for confounding variables (see model 3 in Supplemental Digital Content 1, http://links.lww.com/PSYMED/A753) and subset sensitivity analyses, effects were largely unchanged. Figure 2 shows the model 2 (unadjusted) EMMs over the phases of treatment (residential ➔ partial hospital ➔ intensive outpatient), and Table 2 shows the adjusted EMMs over the treatment phases. Supplemental Digital Contents 2 (http://links.lww.com/PSYMED/A754) and 3 (http://links.lww.com/PSYMED/A755) provide the adjusted EMMs in a table and figure for the individual diagnostic categories.

**TABLE 2:** Confounder-Adjusted EM Mean Global EDE-Q Scores

Treatment Stage	Patient Attributed Eating Disorder Onset to Anti-obesity Messaging						
		Yes (*n* = 522)		No (*n* = 1310)		Unsure (*n* = 1053)	
	Day	EM Mean	95% CI	EM Mean	95% CI	EM Mean	95% CI
RTC admission	0	3.22*^a^*	2.98–3.47	2.87*^b^*	2.64–3.09	3.00*^c^*	2.77–3.23
RTC discharge	38	1.92*^d^*	1.67–2.17	1.67*^e^*	1.45–1.90	1.81*^e^*	1.58–2.05
PHP discharge	73	1.64*^f^*	1.38–1.91	1.50*^f^*	1.26–1.74	1.65*^f^*	1.40–1.90
IOP discharge	119	1.29*^g^*	0.92–1.67	1.29*^g^*	0.94–1.63	1.44*^g^*	1.08–1.80

Days represent median discharge times for patients enrolled in all three levels of care. EM = estimated marginal; EDE-Q = Eating Disorder Examination Questionnaire; CI = confidence interval; RTC = residential treatment center; PHP = partial hospital program; IOP = intensive outpatient program.

Differing subscripts across rows denote statistically significant differences between the anti-obesity messaging trigger subgroups (*p* < .05). Covariates in the model included age, gender identity, race, months since ED onset, prior ED treatment, percent target body weight at intake, trauma history, sexual abuse history, and bullying history.

## DISCUSSION

Anti-obesity messages are a ubiquitous phenomenon targeting individuals who are higher on the weight spectrum, a group showing elevated rates of EDs in recent decades (8,78). To our knowledge, there is no prior research on how common it is for individuals receiving ED treatment to attribute the onset of their ED behaviors to anti-obesity messaging, nor is there previous research identifying the most frequently recollected sources of such messaging. This study is the first to investigate how male- and female-identifying patients attributing their ED onset to anti-obesity messaging compare with their peers without an anti-obesity messaging trigger on ED symptom severity at treatment admission and discharge and on improvement rates over treatment phases.

To summarize, our study found that 18% of patients who received treatment in higher levels of ED specialty care at a US-based center attributed their ED onset to anti-obesity messaging (with another 37% of patients being unsure if anti-obesity messaging precipitated their ED). Perhaps because of the younger age range of this sample, approximately 46% of those patients recalled that the source of the anti-obesity message(s) was the educational curriculum and school context. Internet and other media outlets (24.7%), health care providers (10.4%), family comments (9%), and peer bullying (3.7%) were other identified sources.

In addition, we found that this group of patients, at admission and through the initial phase of treatment, was more compromised by ED symptom severity, as measured by global EDE-Q scores, even in fully confounder-adjusted models. By discharge from the program, however, these differences disappeared. That is, during treatment, the symptom gap between patients who attributed the onset of their ED behaviors to anti-obesity messaging and those who did not (or were unsure) entirely diminished (Figure 2). Importantly, the model showed that it was during phase 2 (partial hospital and intensive outpatient) where the differential benefits emerged, eliminating the gap between groups in global EDE-Q scores.

Along with the overall greater rate of change in symptom improvement for patients attributing their ED to anti-obesity messaging during the latter phase of treatment, Figure 2 also shows that, on average, patients from both groups demonstrated a parallel course of change during the initial phase of treatment. This trend means that, although significant improvements were seen in EDE-Q scores at the completion of the first treatment phase, patients attributing their ED to anti-obesity messaging were not able to “catch up” to the lower symptom profile of their peers unless they were also afforded an additional stay in a lower level of care.

Notably, the “catch-up” during the latter phase of treatment for the patients attributing their ED onset to anti-obesity messaging may reflect the trauma-informed and weight-inclusive (Health At Every Size) framework championed in the outpatient facilities of this center (79). This approach rejects socially sanctioned body norms; recognizes structural and institutional weight biases upheld in the culture; and teaches patients to build trust in their body’s internal signals for hunger, satiety, and movement—of critically important value for individuals with EDs who frequently display disruptions in interoceptive awareness (80). Research with nonclinical populations of higher-weight women engaging in disordered eating showed the importance of addressing weight stigma for program effectiveness (81). The present study brings this treatment target to the clinical environment, suggesting favorable results, especially in the partial hospital and intensive outpatient settings. In particular, the finding that patients with the anti-obesity messaging trigger did not reach the lower symptom profile of their peers during the initial phase of treatment implies that a stronger weight-inclusive approach might also benefit the residential treatment milieu (where weight-normative medical models typically have greater influence), specifically patients who have had negative experiences with anti-obesity messaging and/or are higher on the weight spectrum.

As echoed in recent commentary, these data underscore the importance of prioritizing ED prevention in the public health policy agenda and highlight the need for a weight stigma lens when doing so (82,83). The content of school health programs and curricula is often instituted in response to governmental mandates that act on behalf of well-intentioned policymakers and public health advisees. Unfortunately, health-related school programs, such as the BMI report card (legislatively required in half of US states), are often established before evidence of effectiveness—or potential harms—is understood (21–24). Accordingly, we suggest incorporating a weight-inclusive curriculum into health education (79). Removing the focus on weight and instead emphasizing health-affirming self-care behaviors (e.g., good nutrition, moderate physical activity) rooted in trusting the body would likely enhance health and well-being without putting vulnerable students at potential risk for EDs.

There are also clinical and training implications of import here. Our study shows that a subgroup of patients with severe EDs perceive themselves as having been significantly impacted by weight-stigmatizing messages. As such, the findings suggest that the EDs of this subset of patients are precipitated, if not caused, by an iatrogenic sociocultural factor and are at least as severe and clinically relevant as EDs presumably caused by other (e.g., neurodevelopmental) factors. The approaches used at this center seemed to help these patients and warrant further research attention. The findings also highlight the need for educators-, school personnel-, and clinicians-in-training across the health professions to receive weight bias education with an intersectional lens from those with expertise in EDs (84,85).

### Limitations and Strengths

There are several study limitations, including the retrospective methodology and a sample consisting almost entirely of patients who could access care via private insurance. We also used an exposure variable based solely on interpretive recall. Even though clinicians were trained to elicit information in a standardized fashion, patients may have differing levels of insight about their illness onset, as well as a varying understanding of when their ED began. Given our findings, incorporation of a validated weight stigma measure into the treatment intake process would expand on these results by showing whether patients with higher scores on past experiences of weight stigma are more symptomatic at admission and have different trajectories of change. In addition, ED diagnoses were not uniformly determined via a structured diagnostic interview but rather by a referring provider’s diagnosis and the confirmation of a structured intake interview by a clinician. We are also unable to specifically ascertain why the changes in rates of improvement were delayed until the latter phase of treatment for those who attributed their ED onset to anti-obesity messaging without a formal study of treatment processes in the residential versus partial hospital and intensive outpatient programs. Future research should implement prospective, controlled designs with process measures reflecting a weight-inclusive approach—such as internalized weight bias, body appreciation, self-compassion, and interoceptive awareness—to understand mechanisms driving the effects.

The study has a number of strengths as well. These include the use of a multilevel modeling framework allowing for examination of predictors of nonlinear trajectories of change. Also, use of a large, transdiagnostic sample with a wide age range, representation from both female- and male-identifying patients, and multiple racial identities is a strength. Finally, nonacademic multisite ED treatment centers have been criticized for failures to conduct studies of their treatment outcomes; the present research is a step toward addressing this gap (86).

### Concluding Remarks

This study contributes to our understanding of the impact of anti-obesity messaging on patients receiving ED specialty care. Importantly, two commonly cited sources of the anti-obesity messages are trusted institutions (i.e., school and health care systems) and the people therein. Although improvements were achieved for both groups during residential treatment (Figure 2), patients whose EDs were prompted by anti-obesity messaging needed to continue treatment through the lower levels of care to diminish the symptom severity gap evident at treatment admission. Given the emphasis of a weight-inclusive (Health At Every Size) framework in the outpatient settings from which the data were derived, the findings highlight the importance of fully adopting this approach in the residential setting. Indeed, in light of research linking weight stigma to avoidance of preventive care, we recommend weight-inclusive practices for health care more generally and especially for obesity prevention public health campaigns and policymakers attempting to improve population health (51,87). We hope that this research serves as a catalyst for regarding and researching weight-based public health messages with a critical lens. A wide variety of converging data show that improving the health of individuals requires shifting the focus from body size to the larger societal forces dictating access to good nutrition and health care, as well as environments—both physical and social—that support health-promoting behaviors and personal agency. Enhancing nutrition and physical activity for all, irrespective of BMI, should be the centerpiece of public health messaging purported for preventing disease related to higher-weight status (79,88,89). Finally, a purposeful line of inquiry is needed for examining how interventions intended to improve health systemically interact with social structures and norms in ways that ultimately produce unintended negative consequences, especially in the most vulnerable populations (65).

## Supplementary Material

SUPPLEMENTARY MATERIAL
