# Supplementary material for: Treatment Outcomes and Trajectories of Change in Patients Attributing Their Eating Disorder Onset to Anti-obesity Messaging
Source: Psychosom Med. 2021 Jun 19;83(7):777–86. doi: 10.1097/PSY.0000000000000962 (PMC8428859; doi:10.1097/PSY.0000000000000962)
Supplement: SUPPLEMENTARY MATERIAL [file psymed-83-777-s001.docx]

**Title:** Treatment outcomes and trajectories of change in patients attributing their eating disorder onset to anti-obesity messaging

**Short title:** Eating disorders and anti-obesity messaging

**Authors:** Janell L. Mensinger, PhD, FAED^1^, Shelbi A. Cox, BS^2^ Jennifer R. Henretty, PhD^2^

**Affiliations:** ^1^Villanova University, M. Louise Fitzpatrick College of Nursing, Villanova, PA, ^2^Center For Discovery, Discovery Behavioral Health, Los Alamitos, CA

**Corresponding Author:**

Janell L. Mensinger, PhD, Fellow AED, Associate Research Professor, Biostatistician

800 E. Lancaster Ave.

Villanova University

M. Louise Fitzpatrick College of Nursing

Villanova, PA 19185

Contact: (office phone) 610-519-6806 / (email) [janell.mensinger@villanova.edu](mailto:janell.mensinger@villanova.edu)

**Note:** The research began while the first author was an Associate Research Professor and Director of Biostatistics in the Department of Epidemiology and Biostatistics, Dornsife School of Public Health, Drexel University

**Conflicts of Interest and Sources of Funding:** The second and third author are employees of the treatment center from which the data were drawn. The corresponding author was granted funding from the treatment center to design, conceptualize, and conduct the analysis for the reported research.

**Acknowledgments:**  The authors would like to thank Amanda Morris, MS, and Alexander Costello, BA, who served as Graduate Assistants in Applied Statistics for assistance on technical components of the manuscript. We would also like to acknowledge Dr. Craig Brown, founder of Center For Discovery, and the participating clinicians and patients without whom this research would not have been possible.

2 figures; 2 tables; 3 SDC files (.docx)

**Abstract**

**Objective:** Given increased prevalence of eating disorders (EDs) among individuals higher on the weight spectrum we aimed to: 1) report the prevalence of ED patients in higher levels of care (residential, partial hospitalization, and intensive outpatient) attributing the onset of their ED to anti-obesity messaging, 2) report the most commonly recollected sources of those messages, and 3) determine if those attributing the onset of their ED to anti-obesity messaging a) enter, b) exit, and c) respond to treatment differently than peers who did not. **Methods:** This retrospective cohort study utilized data from 2,901 patients receiving ED treatment in higher levels of care at a US-based center between 2015 and 2018. Multi-level models examined differences in ED symptoms and trajectories of change over time. NVivo was used to analyze the patients’ comments about sources of messages. **Results:** 18% attributed their ED onset to anti-obesity messaging, 45% did not, 37% were unsure. Of those providing comments, the most common sources included: educational curriculum/school context (45.9%), media/internet (24.7%), healthcare (10.4%), family (9%), and peer bullying (3.7%). At admission, patients attributing their ED onset to anti-obesity messaging had more severe ED symptoms than those who did not (γ=0.463, SE=0.086, *p*<.001) and those who were unsure (γ=0.288 SE=0.089, *p*<.001); no differences were evident at discharge (*p values* >.483). During phase two of treatment, patients attributing their ED onset to anti-obesity messaging improved faster than those who did not (γ=0.003, SE=0.001, *p=*.008) and those who were unsure (γ=0.003, SE=0.001, *p=*.014). **Conclusion:** Anti-obesity messaging may put vulnerable individuals at risk for EDs. We recommend increasing weight bias training for school personnel and healthcare professionals. To reduce health disparities, we also suggest the promotion of weight-neutral health-enhancing self-care practices in media and public health campaigns, legislative policies, and healthcare overall.

*Key words:* anti-obesity messaging; eating disorders; weight stigma; weight-inclusive care; higher levels of care; eating disorder treatment outcomes

**Abbreviations**

Analysis of Variance: ANOVA

Anorexia Nervosa - Binge/Purge type: AN-B/P

Anorexia Nervosa - Restricting type: AN-R

Anorexia Nervosa: AN

Avoidant/Restrictive Food Intake Disorder: ARFID

Binge Eating Disorder: BED

Body Mass Index: BMI

Bulimia Nervosa: BN

Confidence Interval: CI

Diagnostic and Statistical Manual of Mental Disorders (5th Edition): DSM-5

Eating Disorder Examination-Questionnaire: EDE-Q

Eating Disorder(s): ED(s)

Electronic Medical Records: EMR

Estimated Marginal: EM

Estimated Marginal Mean: EMM

Estimated Marginal Mean Differences: EMM Δ

Institutional Review Board: IRB

Intensive Outpatient Program: IOP

Interquartile Range: IQR

Kilogram: kg

Kilograms/Meters squared: kg/m^2^

Other Specified Feeding and Eating Disorder: OSFED

Partial Hospital Program: PHP

Percent Target Body Weight: %TBW

Residential Treatment Center: RTC

Standard Error: SE

Statistical Analysis System: SAS

Statistical Product and Service Solutions: SPSS

Strengthening the Reporting of Observational Studies in Epidemiology: STROBE

Target Body Weight: TBW

United Kingdom: UK

United States: US

Unspecified Feeding and Eating Disorder: UFED

**Introduction**

Eating disorders (EDs) are serious mental health conditions that cause significant health problems—even death—and early identification and treatment are crucial for best prognosis (1-3). While estimated prevalence rates vary across the globe, one recent study of Australian adolescents showed 22.2% with a probable ED diagnosis from the 5^th^ Edition of the Diagnostic and Statistical Manual of Mental Disorders (DSM-5) (4), including the *unspecified* feeding and EDs (i.e., UFED, syndromes not fitting into the established criteria for specified disorders) (5). A recent systematic review of global prevalence rates demonstrated a doubling of EDs point prevalence since the early 2000’s (from 3.5% to 7.8%, across all ages) (6). Furthermore, several epidemiological studies show alarming increases in disordered eating among people with body mass index (BMI) over 30 kg/m^2^ (7-9). Though these increases may be in part due to new attention on the historically under recognized restrictive EDs in higher weight people (10, 11), some researchers speculate that these increases are a consequence of widespread public health campaigns around the dangers and problems related to high BMI (12-15).

Hunger and Tomiyama recently used modified labeling theory to investigate the potential harm in messages about weight (16). The approach posits that terms used to describe an individual will contribute to their identity development and influence future behavior. More specifically, modified labeling theory suggests that the damaging and stigmatizing effects of a label occur because, no matter how well-intentioned the act, identifying someone as a member of any socially devalued group often leads to internalization of negative societal stereotypes about the group (17). Consistent with this theory, their study demonstrated that classifying an adolescent girl as “too fat” was associated with greater disordered behaviors and cognitions five years later, and this effect was especially pronounced when the source of the labeling was a family member (16). Indeed, family members have been cited as the most common source of weight stigma, followed by doctors and then classmates (18).

Though identification of a child’s elevated weight status is traditionally considered an important method of obesity prevention, research has shown insufficient data to recommend for or against BMI surveillance for youth (19-21). In fact, a recent large cluster randomized clinical trial of 28,641 California students in grades 3 through 8 found no changes in BMI z-scores among higher weight students (>85^th^ percentile) in schools implementing the BMI screening protocol (22). Moreover, compared to the control group students (i.e., schools with no BMI surveillance protocol), body dissatisfaction and peer weight talk increased significantly more among students in the schools that were assigned the BMI screening (22).

As modified labeling theory implies, other studies also suggest weight surveillance may come with unintended consequences, especially if not implemented with the Center for Disease Control’s recommended safeguards, which few schools do (23-25). Using an econometric model allowing for causal inference, a study of the New York City school system showed girls given the “overweight” label in their BMI report card had significantly greater BMI gains the following year compared to the control group girls not labeled (26). Though the negative effect was small overall, it became more pronounced for older girls (i.e., those in their junior year), and for those never previously labeled “overweight.” There was no labeling effect for boys (26).

In related research, adolescent girls participating in the National Heart Lung and Blood Institute’s Growth and Health Study who reported being identified as “too fat” also had significantly higher odds of an “obese” BMI 10 years later, even after adjusting for baseline BMI, race, income, and parental education (27). Similarly, adolescents with BMIs in the “overweight” and “obese” range from the National Longitudinal Study of Adolescent to Adult Health wave II cohort were followed to determine how their perceived weight influenced future weight change (28). Those who *inaccurately* described themselves as having a “normal” weight had significantly *lower* BMI gains after 13 years, even after adjusting for baseline BMI (28). In a connected line of work, Schvey and colleagues showed that perceived pressures to be thin were associated with insulin resistance in adolescent boys and girls, and the relationships were maintained when adjusting for body composition (i.e., % lean mass and kg of fat mass) (29).

Comparable associations hold in adult samples. Perceiving oneself as “overweight,” whether the perception was medically accurate or not, predicted future weight gain in over 14,000 adults from the US and UK, which again, held true after baseline BMI adjustment (30). In addition, a study of 3,582 adults in the US implied that messages warning individuals of their “weight problems” do not predict health improvements in the long run (31). To the contrary, researchers found that perceptions of being “overweight,” irrespective of actual BMI at baseline, were associated with worse long-term physiological dysregulation and poorer subjective health ratings after a 7-year period compared to those without such perceptions (31). In a nationally representative sample of 6,157 adults, those reporting weight-based discrimination were also more likely to have increased their weight by follow-up, adjusting for baseline BMI, age, sex, ethnicity, and education (32). In sum, through multiple samples and methodologies, these studies show the ineffectiveness and potential harm of labeling strategies for higher weight individuals.

Multiple studies also experimentally demonstrated the dangers of messages about food and body in laboratory environments. For instance, after exposure to dieting products and slender models, self-identified restrained eaters further restricted food intake (33). Likewise, body dissatisfaction increased after viewing “thin-and-beautiful” media images (34). Moreover, Puhl et al. showed that stigmatizing ads induced less self-efficacy for change in health-related behaviors than neutral messages in a large randomized trial of the American public’s (*n*=1085) reactions to weight-related health campaigns (35). This finding supports earlier research suggesting weight stigma is associated with exercise avoidance, as well as a whole host of negative sequelae—physiological, psychological, and behavioral (36, 37). It also sheds light on the problem that, per a recent review , 44% of obesity-related public health campaigns included a stigmatizing strategy, even though research shows people evaluate messages as more helpful and motivating when they are *not* stigmatizing—or weight-based at all—but rather focus solely on healthy behaviors (15, 38).

This research underpins cross-sectional evidence supporting an expanded tripartite influence model of ED risk whereby family, peer, partner, and media pressures about weight are associated with disordered eating through the internalization of the thin-ideal, and over a decade of studies showing that internalized and experienced weight stigma are related to disordered eating (39-43).

Finally, though it is well-known that EDs have a multifactorial etiology involving an interplay of psychosocial, environmental, and genetic risk (44), dieting behaviors—especially extreme weight control practices (e.g., fasting, diet pills, laxative use)—have long been conceptualized as fundamental precursors for EDs (45). While the relationship is nuanced (i.e., not all diets lead to EDs) (46), this link has been established through experimental frameworks, as shown in restraint theory (47, 48), and through longitudinal research on the relationship between early weight-control behaviors and the later development of disordered eating and/or diagnosed clinical EDs (49, 50).

Thus, a large number and wide variety of studies raise critical concerns surrounding the ethics of using weight-related public health messaging (51). Considering weight loss interventions do not consistently lead to the oft-presumed health benefits (52-57), particularly in the long term (58), and given that dieting is also associated with weight cycling, which escalates both morbidity and mortality (59-63), we must exercise caution with the use of anti-obesity public health messaging.

The present research seeks to underscore the need for attention to the potential harm of weight-based public health interventions, and the consequential trickle-down effects of these interventions—from government mandated policy change of the public-school health curriculum to advertising campaigns, and even one-on-one individualized messaging (e.g., from well-meaning healthcare providers and family members). Health researchers have long warned of iatrogenic effects of public health interventions, particularly towards prevention of “obesity” in children (23-25, 64). Though Bonell and colleagues have theorized about methods for uncovering mechanisms of harm in public health interventions, such theory is rarely evaluated (65). Our goal is to investigate one component of this matter in a large sample of patients in treatment for an ED by documenting reports of anti-obesity messages as the factor prompting the onset of their ED.

Despite the widespread knowledge that socio-cultural pressures toward thinner body norms are among the most robust risk factors of EDs (44), to our knowledge no previous studies have examined how often patients entering treatment for an ED attribute the onset of their disorder to anti-obesity messaging, and whether those who do are any more or less symptomatic at treatment admission and discharge than their peers whose EDs were not triggered by anti-obesity messaging. Therefore, the specific aims of this study are to: 1) report the prevalence of ED patients in higher levels of care (i.e., residential, partial hospital program, and intensive outpatient treatment) attributing *the onset* of their ED to anti-obesity messaging, 2) report the most commonly recollected sources of those messages, and 3) determine if patients attributing the onset of their ED to anti-obesity messaging a) enter, and b) exit treatment with more or less severe symptoms, and c) respond to treatment at a faster or slower rate than peers who did not attribute the onset of their ED to anti-obesity messages.

**Methods**

**Participants and Setting**

The study was a retrospective cohort design of 2,901 patients receiving treatment for a diagnosed ED at a US-based ED specialty center. ED diagnosis was given by the referring clinician and confirmed by the intake clinician upon admission to treatment. The American Psychiatric Association’s Practice Guidelines for the Treatment of Patients with EDs (66) along with the medical necessity criteria defined by the third-party insurance payors were used for determination of eligibility into the treatment settings. Diagnoses ranged across the full spectrum of DSM-5 EDs: 1) Anorexia Nervosa-Restricting type (AN-R); 2) Anorexia Nervosa-Binge/Purging type (AN-B/P); 3) Bulimia Nervosa (BN); and 4) Binge Eating Disorder (BED); 5) Avoidant/Restrictive Food Intake Disorder (ARFID); and 6) Other Specified Feeding And Eating Disorder (OSFED) a category that includes (a) Purging Disorder; (b) Atypical Anorexia Nervosa (i.e., when all features of AN are met except that despite weight loss, the individual’s weight remains within or above a “normal” range), (c) Night Eating Syndrome, (d) low frequency or short duration BN, and (e) low frequency or short duration BED.

We extracted deidentified data from the center’s electronic medical records (EMR) based on the inclusion criteria of the first and most complete, contiguous treatment involving a downward progression in intensity of care (i.e., residential treatment🡪partial hospital🡪intensive outpatient) whereby patients were discharged to the next lower level of care as appropriate (and/or determined by insurance), between the years of 2015 and 2018. These criteria ensured there were no repeated cases in the dataset. To best assure available data for longitudinal analysis, we excluded a patient’s first episode if a later episode was more intensive and involved moving through multiple levels of care. All patients included in the database had consented to have their information available for future research studies (see Figure 1 for the STROBE flow diagram). The Drexel University Institutional Review Board (IRB) reviewed the protocol and provided a letter of determination that the research was considered “exempt.”

Most of the sample (*n*=2,094, 72%) was admitted to residential treatment (24-hour care); 478 (16%) initiated treatment in a partial hospital program (typically 5-6 days/week, 8 hours/day) and 329 (11%) began in intensive outpatient (typically 3-4 days/week, 4 hours/day). The final person-period dataset included 7,663 observations: 2,061 had one level of care consisting of admission and discharge (79% of which were residential), 565 people had two levels of care consisting of two admissions and two step-downs (65% of which were partial hospitalization to intensive outpatient), and 275 had all levels of care encompassing three admissions and three step-downs.

**Procedures for variable measurement**

Variables drawn from the EMR extraction included age, gender identity, race/ethnicity, ED diagnosis, and percent target body weight (%TBW) at admission. Percent TBW was determined by the Hamwi method for adults (<http://www.scymed.com/en/smnxpn/pndfc237.htm>) and the following formula for adolescents (<18 years): (50th percentile BMI*height in inches^2^)*(2.2). We collected %TBW as opposed to BMI, given the large proportion of adolescent patients in the sample; BMI is a less reliable measurement for capturing weight-to-height ratio in adolescence. The correlation between BMI and %TBW was .99 in the current dataset. As part of the standard, structured biopsychosocial intake completed by a licensed therapist (or under the supervision of one), patients were asked about the months since the onset of their ED; prior ED treatment; and history of bullying, sexual abuse, and other forms of trauma (e.g., physical, emotional, and verbal abuse—which were combined to form a single “other trauma” variable).

The study’s primary exposure variable—whether the ED onset was attributed to anti-obesity messaging—was charted by the intake clinician. Clinicians were trained to perform all questioning and response coding for the structured biopsychosocial intake in a standardized format. After being asked how old they were when their ED started, patients were next asked *“Can you remember if there was something that happened that led to the start of the ED.”* If the response involved an anti-obesity messaging trigger, the exposure was coded as *‘yes’*, and the clinician probed further by asking “*What was the primary source of those messages?”* Patients who were unsure of what triggered their ED received an *‘unsure’* for the anti-obesity messaging variable. Patients naming other factors leading to their ED received a *‘no.’*

The Eating Disorder Examination-Questionnaire (EDE-Q) was completed at admission and discharge from each level of care as a measure of treatment success (67). The EDE-Q is a well-validated, self-report measure of ED severity that is among the most widely adopted transdiagnostic tools to assess ED treatment outcomes (68). It has established normative data for clinical populations, and it has been shown to be responsive to treatment effects (69-71). The EDE-Q asks patients to evaluate the extent to which they have experienced cognitive and behavioral components of EDs over the past 28 days on a scale from 0 (*no days*) to 6 (*every day*). Global scores were calculated as the study outcome variable; higher scores indicate greater symptom severity.

The maximum number of timepoints per participant was six—an admission and discharge for each level of care. About three-quarters of the sample were initially admitted into residential treatment. However, given that some patients began treatment in partial hospital or intensive outpatient, reducing the number of timepoints available for them, we created an indicator variable for level of care admission to allow adjustment for this in all models. The time variable utilized for the models presented is the exact day of assessment, counting from initial admission as day 0 to each subsequent discharge and/or stepdown-admission assessment.

**Data Analysis**

Data were imported into SPSS (version 25) and cleaned and analyzed using SPSS and SAS (version 9.4). Exploratory analyses included examining variable distributions with stem-and-leaf and box-and-whisker plots to detect outliers and distribution problems. Number of months since ED onset was log transformed to normalize right skewness. Model diagnostics were performed by plotting the predicted against observed values and examining multivariate normality with histograms of the residuals. We also plotted the intercepts and slopes to establish that multivariate normality was met for the random coefficients portion of the models. Model-based estimates showed excellent fit to the observed data. One-way ANOVAs, chi-squares, and Kruskal Wallis ANOVAs were applied, as appropriate, to show whether there were differences on the covariates between patients attributing their ED onset to anti-obesity messaging and those who did not or were unsure. Bivariate Pearson correlations between the continuous covariates were examined for potential multicollinearity problems. Given the high correlation between months since ED onset and age (*r*=.84), we created a dichotomized age variable (<19 years).

To answer Aim 1, frequencies and proportions were calculated to determine the prevalence of attributing anti-obesity messages to ED onset. For Aim 2, we used thematic content analysis (72) initiated by a word frequency query in NVivo to identify content themes on the descriptive responses of the source of the anti-obesity messages. Subsequent text search queries were run on the most frequently occurring words identified in the responses. The process was completed by combining themes into categories (e.g., health class, physical education, teacher comments, and cooking class were combined into a single broad category to capture the educational curriculum and school environment).

Aim 3 was answered using multilevel mixed-effects models, which examined subject-specific differences in symptom severity (a) at initial admission, (b) at discharge, and (c) trajectories of symptom change through treatment. In order to determine the best fitting function(s) for time (Aim 3c), empirical Bayes plots were fit to a random set of 50 observations to detect underlying trends for the trajectory of symptom change. Combining this information as an initial guide indicating nonlinear change, in accordance with practices consistent with Singer and Willet’s framework for applied longitudinal analysis (73), we fit a series of unconditional growth models using various nonlinear functions of time to best represent trajectories of change through treatment. The final unconditional growth model (plus adjustments for initiating level of care; Supplemental Digital Content 1, <http://links.lww.com/PSYMED/A753>, Model 1) was chosen using the likelihood ratio test and examining Akaike’s criterion for the best model fit.

The best fitting model included a random intercept function, which captures person-specific initial status of EDE-Q at intake, and two random slope functions (a reciprocal function of time and linear time function) to represent the person-specific modeling of initial acceleration of change in symptoms at the beginning of treatment (reciprocal function) and a flatter change (linear function) captured during the second half of treatment. Subsequent models were fit to answer Aim 3 without adjustments (Model 2) and with confounder adjustments (Models 3). The confounder-adjusted model shows the primary exposure variable of interest (anti-obesity messaging), plus the following *a priori* covariates: age; gender identity; race/ethnicity; ED diagnosis; prior treatment; %TBW at admission; and history of sexual abuse, bullying, and other trauma—all chosen based on theory and past research in the ED field about the importance of severity markers (e.g., length of illness, prior treatment), demographics (e.g., age, race, gender identity), and abuse-related risk factor differences (70, 74-77). To preserve degrees of freedom and maintain a parsimonious model, covariates not reaching *p*<.10 were dropped; all covariates remained on at least one of the model levels (i.e., initial status, slope during phase 1, or slope during phase 2). Models are presented per Singer and Willet (73) style in Supplemental Digital Content 1, http://links.lww.com/PSYMED/A753.

Because multilevel models use maximum likelihood estimation, which keeps the participant in the analysis as long as they have at least one observed outcome (all 2,901 participants had a minimum of 2 timepoints), only missing values on covariates impacted the models. Given this problem was minimal (see available *n*’s in Table 1), we were able to use over 95% of the full sample. A sensitivity analyses was conducted on the complete sample (minus the 16 cases missing on the anti-obesity messaging trigger, 0.55%) for Model 2, which did not include covariates, to confirm robustness of the results regarding the primary exposure variable. Multiple sensitivity analyses with subsets of the data were also performed.

**Results**

Table 1 displays the patient characteristics broken down by whether they attributed their ED onset to anti-obesity messaging (yes, no, unsure). The sample had a mean age of 21.7 (*SD*=7.32, range 9-83). Due to the age distribution’s extreme right skew, in Table 1 we present the median (Interquartile Range, IQR) of 17 years (15-23). The large majority of the sample was female-identifying (94%, *n*=2,708) and the remaining 6% (*n*=177) was male-identifying; 0.5% (*n*=16) had no response to the gender identity question. The most frequent ED diagnosis was anorexia nervosa-restricting type (43%; *n*=1240), 19% (*n*=532) had a diagnosis of bulimia nervosa, 17% (*n*=495) anorexia nervosa-binge/purging type, 12% (*n*=336) other specified feeding and eating disorder, 7% (*n*=197) binge eating disorder, and 3% (*n*=83) were diagnosed with avoidant/restrictive food intake disorder. Most patients (78%; *n*=2,165) identified as White non-Hispanic, 12% (*n*=338) as Latinx, 4% (*n*=124) as Asian or Pacific Islander, 2% (*n*=49) as African American or Black, 1% (*n=*15) as American Indian or Native Alaskan, and 3% (*n*=95) identified as multiple races/ethnicities. No racial/ethnic identity information was provided for 115 patients.

Of the 2,901 patients, 522 (18%) attributed their ED onset to anti-obesity messaging, 1,053 (37%) were unsure if anti-obesity messaging precipitated their ED, and the remaining 1,310 (45%) attributed it to other factors. Content analysis of the descriptive data showed that the most commonly recollected source of anti-obesity messages in this patient sample was related to the theme of education curriculum and school environment, (45.9% of the 490 patients who named sources of anti-obesity messages). Sources of messaging from the educational curriculum theme included reference to classes and teachers in general, as well as health, nutrition, cooking, and, physical education classes more specifically. The second most common source of messaging came from the internet/social media and general media (24.7%). Other themes included messaging from healthcare providers (10.4%), family members (9%), and peer bullying (3.7%). Not all sources (e.g., church) fell under identified themes.

Patients who attributed their ED onset to anti-obesity messaging had significantly higher EDE-Q scores at admission than those who did not and those who were unsure, with estimated marginal mean differences (EMM Δ) of 0.463 [95% CI=0.295, 0.631] and 0.288 [95% CI=0.114, 0.462], respectively. By final discharge, however, these differences were no longer evident for the “yes” versus “no” groups (EMM Δ=0.062, 95% CI=-0.197, 0.321) or the “yes” versus “unsure” groups (EMM Δ=-0.096, 95% CI=-0.366, 0.173). Tests of differences in rates of change over the first phase of treatment (captured in the reciprocal time trend) showed that patients attributing their ED onset to the anti-obesity messaging were no different from their peers who did not (γ=-0.008, SE=0.124, *p=*.950) or their peers who were unsure (γ=0.062, SE=0.128, *p=*.625), meaning when patients with the anti-obesity message trigger were discharged from the *initial* phase of treatment, they remained more symptomatic than their peers without the trigger. However, during the *latter* phase of care (captured in the linear time trend), patients attributing their ED onset to anti-obesity messaging improved significantly faster than those who did not (γ=0.003, SE=0.001, *p=*.008) and those who were unsure (γ=0.003, SE=0.001, *p=*.014), showing final discharge at equal levels of symptom severity. After adjusting for confounding variables (see Model 3 in SDC 1) and subset sensitivity analyses, effects were largely unchanged. Figure 2 shows the Model 2 (unadjusted) EMMs over the phases of treatment (residential🡪partial hospital🡪intensive outpatient), and Table 2 shows the adjusted EMMs over the treatment phases. Supplemental Digital Content 2, http://links.lww.com/PSYMED/A754 and 3, http://links.lww.com/PSYMED/A755 provide the adjusted EMMs in a table and figure for the individual diagnostic categories.

**Discussion**

Anti-obesity messages are a ubiquitous phenomenon targeting individuals who are higher on the weight spectrum—a group showing elevated rates of EDs in recent decades (8, 78). To our knowledge, there is no prior research on how common it is for individuals receiving ED treatment to attribute the onset of their ED behaviors to anti-obesity messaging, nor is there previous research identifying the most frequently recollected sources of such messaging. This study is the first to investigate how male and female-identifying patients attributing their ED onset to anti-obesity messaging compare to their peers without an anti-obesity messaging trigger on ED symptom severity at treatment admission and discharge and on improvement rates over treatment phases.

To summarize, our study found that 18% of patients who received treatment in higher levels of ED specialty care at a US-based center attributed their ED onset to anti-obesity messaging (with another 37% of patients being unsure if anti-obesity messaging precipitated their ED). Perhaps due to the younger age range of this sample, approximately 46% of those patients recalled that the source of the anti-obesity message(s) was the educational curriculum and school context. Internet and other media outlets (24.7%), healthcare providers (10.4%), family comments (9%), and peer bullying (3.7%) were other identified sources.

Additionally, we found that this group of patients, at admission and through the initial phase of treatment, was more compromised by ED symptom severity, as measured by global EDE-Q scores, even in fully confounder-adjusted models. By discharge from the program, however, these differences disappeared. That is, during treatment, the symptom gap between patients who attributed the onset of their ED behaviors to anti-obesity messaging and those who did not (or were unsure) entirely diminished (see Figure 2). Importantly, the model showed that it was during phase 2 (partial hospital and intensive outpatient) where the differential benefits emerged, eliminating the gap between groups in global EDE-Q scores.

Along with the overall greater rate of change in symptom improvement for patients attributing their ED to anti-obesity messaging during the latter phase of treatment, Figure 2 also shows that, on average, patients from both groups demonstrated a parallel course of change during the initial phase of treatment. This trend means that, though significant improvements were seen in EDE-Q scores at the completion of the first treatment phase, patients attributing their ED to anti-obesity messaging were not able to “catch up” to the lower symptom profile of their peers unless they were also afforded an additional stay in a lower level of care.

Notably, the “catch up” during the latter phase of treatment for the patients attributing their ED onset to anti-obesity messaging may reflect the trauma-informed and weight-inclusive (Health At Every Size®) framework championed in the outpatient facilities of this center (79). This approach rejects socially sanctioned body norms; recognizes structural and institutional weight biases upheld in the culture; and teaches patients to build trust in their body’s internal signals for hunger, satiety, and movement—of critically important value for individuals with EDs who frequently display disruptions in interoceptive awareness (80). Research with non-clinical populations of higher weight women engaging in disordered eating showed the importance of addressing weight stigma for program effectiveness (81). The present study brings this treatment target to the clinical environment, suggesting favorable results—especially in the partial hospital and intensive outpatient settings. In particular, the finding that patients with the anti-obesity messaging trigger did not reach the lower symptom profile of their peers during the initial phase of treatment implies a stronger weight-inclusive approach might also benefit the residential treatment milieu (where weight-normative medical models typically have greater influence), specifically patients who have had negative experiences with anti-obesity messaging and/or are higher on the weight spectrum.

As echoed in recent commentary, these data underscore the importance of prioritizing ED prevention in the public health policy agenda and highlight the need for a weight stigma lens when doing so (82, 83). The content of school health programs and curricula are often instituted in response to governmental mandates that act on behalf of well-intentioned policymakers and public health advisees. Unfortunately, health-related school programs, such as the BMI report card (legislatively required in half of US states), are often established prior to evidence of effectiveness—or potential harms—are understood (21-24). Accordingly, we suggest incorporating a weight-inclusive curriculum into health education (79). Removing the focus on weight and instead emphasizing health-affirming self-care behaviors (e.g., good nutrition, moderate physical activity) rooted in trusting the body would likely enhance health and well-being without putting vulnerable students at potential risk for EDs.

There are also clinical and training implications of import here. Our study shows that a subgroup of patients with severe EDs perceive themselves as having been significantly impacted by weight-stigmatizing messages. As such, the findings suggest that the EDs of this subset of patients are precipitated, if not caused, by an iatrogenic sociocultural factor, and are at least as severe and clinically relevant as EDs presumably caused by other (e.g., neurodevelopmental) factors. The approaches used at this center appeared to help these patients and warrant further research attention. The findings also highlight the need for educators-, school personnel-, and clinicians-in-training *across the health professions* to receive weight bias education with an intersectional lens from those *with expertise in eating disorders* (84, 85).

**Limitations and Strengths**

There are several study limitations, including the retrospective methodology and a sample consisting almost entirely of patients who could access care via private insurance. We also used an exposure variable based solely on interpretive recall. Even though clinicians were trained to elicit information in a standardized fashion, patients may have differing levels of insight about their illness onset, as well as a varying understanding of when their ED began. Given our findings, incorporation of a validated weight stigma measure into the treatment intake process would expand on these results by showing whether patients with higher scores on past experiences of weight stigma are more symptomatic at admission and have different trajectories of change. Additionally, ED diagnoses were not uniformly determined via a structured diagnostic interview but rather by a referring provider’s diagnosis and the confirmation of a structured intake interview by a clinician. We are also unable to specifically ascertain *why* the changes in rates of improvement were delayed until the latter phase of treatment for those who attributed their ED onset to anti-obesity messaging without a formal study of treatment processes in the residential versus partial hospital and intensive outpatient programs. Future research should implement prospective, controlled designs with process measures reflecting a weight-inclusive approach—such as internalized weight bias, body appreciation, self-compassion, and interoceptive awareness—in order to understand mechanisms driving the effects.

The study has a number of strengths as well. These include the use of a multilevel modeling framework allowing for examination of predictors of nonlinear trajectories of change. Also, use of a large, transdiagnostic sample with a wide age range, representation from both female- and male-identifying patients, and multiple racial-identities is a strength. Finally, non-academic multisite ED treatment centers have been criticized for failures to conduct studies of their treatment outcomes; the present research is a step towards addressing this gap (86).

**Concluding Remarks**

This study contributes to our understanding of the impact of anti-obesity messaging on patients receiving ED specialty care. Importantly, two commonly cited sources of the anti-obesity messages are trusted institutions (i.e., school and healthcare systems) and the people therein. Though improvements were achieved for both groups during residential treatment (see Figure 2), patients whose EDs were prompted by anti-obesity messaging needed to continue treatment through the lower levels of care to diminish the symptom severity gap evident at treatment admission. Given the emphasis of a weight-inclusive (Health At Every Size®) framework in the outpatient settings from which the data were derived, the findings highlight the importance of fully adopting this approach in the residential setting. Indeed, in light of research linking weight stigma to avoidance of preventive care, we recommend weight-inclusive practices for healthcare more generally, and especially for obesity prevention public health campaigns and policymakers attempting to improve population health (51, 87). We hope that this research serves as a catalyst for regarding and researching weight-based public health messages with a critical lens. A wide variety of converging data show that improving the health of individuals requires shifting the focus from body size to the larger societal forces dictating access to good nutrition and healthcare, as well as environments—both physical and social—that support health-promoting behaviors and personal agency. Enhancing nutrition and physical activity for all, *irrespective of BMI*, should be the centerpiece of public health messaging purported for preventing disease related to higher weight status (79, 88, 89). Finally, a purposeful line of inquiry is needed for examining how interventions intended to improve health systemically interact with social structures and norms in ways that ultimately produce unintended negative consequences, especially in the most vulnerable populations (65).

**References**

1. Steinhausen H-C. Outcome of eating disorders. Child Adolesc Psychiatr Clin N Am 2009;18:225-42.

2. O’Brien K, Whelan DR, Sandler DP, Hall JE, Weinberg CR. Predictors and long-term health outcomes of eating disorders. PLoS One 2017;12:e0181104.

3. Treasure J, Russell G. The case for early intervention in anorexia nervosa: theoretical exploration of maintaining factors. Br J Psychiatry 2011;199:5-7.

4. American Psychiatric Association. Diagnostic and statistical manual of mental disorders: DSM-5. 5th ed. Arlington: American Psychiatric Association; 2013.

5. Mitchison D, Mond J, Bussey K, Griffiths S, Trompeter N, Lonergan A, Pike, KM, Murray, Hay P. DSM-5 full syndrome, other specified, and unspecified eating disorders in Australian adolescents: prevalence and clinical significance. Psychol Med 2019;50:981-990.

6. Galmiche M, Déchelotte P, Lambert G, Tavolacci MP. Prevalence of eating disorders over the 2000–2018 period: a systematic literature review. Am J Clin Nutr 2019;109:1402-13.

7. da Luz FQ, Sainsbury A, Mannan H, Touyz S. Prevalence of obesity and comorbid eating disorder behaviors in South Australia from 1995 to 2015. Int J Obes 2017;41:1148-53.

8. Darby A, Hay P, Mond J, Quirk F, Buettner P, Kennedy L. The rising prevalence of co-morbid obesity and eating disorder behaviours from 1995 to 2005. Int J Eat Disord 2009;42:104-8.

9. Duncan AE, Ziobrowski H, Nicol G. The prevalence of past 12‐Month and lifetime DSM‐IV eating disorders by BMI category in US men and women. Eur Eat Disord Rev 2017;25:165-71.

10. Neumark-Sztainer D. Higher weight status and restrictive eating disorders: an overlooked concern. J Adolesc Health 2015;56:1-2.

11. Harrop EN. Typical-atypical interactions: one patient’s experience of weight bias in an inpatient eating disorder treatment setting. Women Ther 2018:1-14.

12. Greenhalgh S. Disordered eating/eating disorder: hidden perils of the nation's fight against fat. Med Anthropol Q 2016;30:545-62.

13. Greenhalgh S. Weighty subjects: The biopolitics of the U.S. war on fat. Am Ethnol 2012;39:471-87.

14. Bristow C, Meurer C, Simmonds J, Snell T. Anti-obesity public health messages and risk factors for disordered eating: a systematic review. Health Promot Int 2020.

15. Turner MM, Ford L, Somerville V, Javellana D, Day KR, Lapinski MK. The use of stigmatizing messaging in anti-obesity communications campaigns: quantification of obesity stigmatization. Communication Reports 2020;33:107-20.

16. Hunger JM, Tomiyama AJ. Weight labeling and disordered eating among adolescent girls: longitudinal evidence from the National Heart, Lung, and Blood Institute Growth and Health Study. J Adolesc Health 2018;63:360-2.

17. Link BG, Cullen FT, Struening E, Shrout PE, Dohrenwend BP. A modified labeling theory approach to mental disorders: an empirical assessment. Am Sociol Rev 1989:400-23.

18. Puhl RM, Brownell KD. Confronting and coping with weight stigma: an investigation of overweight and obese adults. Obesity 2006;14:1802-15.

19. US Preventive Services Task Force. Screening for obesity in children and adolescents. JAMA 2017;317:2417-26.

20. Krebs NF, Jacobson MS, American Academy of Pediatrics Committee on Nutrition. Prevention of pediatric overweight and obesity. Pediatrics 2003;112:424-30.

21. Thompson HR, Madsen KA. The report card on BMI report cards. Curr Obes Rep 2017;6(2):163-7.

22. Madsen KA, Thompson HR, Linchey J, Ritchie LD, Gupta S, Neumark-Sztainer D, Crawford, PB, McCulloch, CE, & Ibarra-Castro, A. Effect of School-Based Body Mass Index Reporting in California Public Schools: A Randomized Clinical Trial. JAMA Pediatr. 2020; Online ahead of print:e204768.

23. Portilla MG. Body mass index reporting through the school system: potential harm. J Am Diet Assoc 2011;111:442-5.

24. Ikeda JP, Crawford PB, Woodward-Lopez G. BMI screening in schools: helpful or harmful. Health Educ Res 2006;21:761-9.

25. Sliwa SA, Brener ND, Lundeen EA, Lee SM. Do schools that screen for body mass index have recommended safeguards in place? J Sch Nurs 2019;35(4):299-308.

26. Almond D, Lee A, Schwartz AE. Impacts of classifying New York City students as overweight. Proc Natl Acad Sci USA 2016;113:3488-91.

27. Hunger JM, Tomiyama A. Weight labeling and obesity: A longitudinal study of girls aged 10 to 19 years. JAMA Pediatrics 2014;168:579-80.

28. Sonneville KR, Thurston IB, Milliren CE, Kamody RC, Gooding HC, Richmond TK. Helpful or harmful? Prospective association between weight misperception and weight gain among overweight and obese adolescents and young adults. Int J Obes 2016;40:328-32.

29. Schvey NA, Shomaker LB, Kelly NR, Pickworth CK, Cassidy O, Galescu O, Demidowich, AP, Brady, SM, Tanofsky-Kraff, M, Yanovski JA. Pressure to be thin and insulin sensitivity among adolescents. J Adolesc Health 2016;58:104-10.

30. Robinson E, Hunger JM, Daly M. Perceived weight status and risk of weight gain across life in US and UK adults. Int J Obes 2015;39:1721-26.

31. Daly M, Robinson E, Sutin AR. Does knowing hurt? perceiving oneself as overweight predicts future physical health and well-being. Psychol Sci 2017;28:872-81.

32. Sutin AR, Terracciano A. Perceived weight discrimination and obesity. PLoS One 2013;8:e70048.

33. Anschutz DJ, Engels RCME, Becker ES, van Strien T. The bold and the beautiful. Influence of body size of televised media models on body dissatisfaction and actual food intake. Appetite 2008;51:530-7.

34. Yamamiya Y, Cash TF, Melnyk SE, Posavac HD, Posavac SS. Women's exposure to thin-and-beautiful media images: body image effects of media-ideal internalization and impact-reduction interventions. Body Image 2005;2:74-80.

35. Puhl R, Luedicke J, Peterson JL. Public reactions to obesity-related health campaigns: a randomized controlled trial. Am J Prev Med 2013;45:36-48.

36. Puhl R, Suh Y. Health consequences of weight stigma: implications for obesity prevention and treatment. Curr Obes Rep 2015;4:182-90.

37. Vartanian LR, Shaprow JG. Effects of weight stigma on exercise motivation and behavior: a preliminary investigation among college-aged females. J Health Psychol 2008;13:131-38.

38. Puhl R, Peterson JL, Luedicke J. Fighting obesity or obese persons? Public perceptions of obesity-related health messages. Int J Obes (Lond) 2013;37:774-82.

39. Wang Z, Wang B, Hu Y, Cheng L, Zhang S, Chen Y, Li R. Relationships among weight stigma, eating behaviors and stress in adolescents in Wuhan, China. Glob Health Res and Policy 2020;5:8.

40. O'Hara L, Tahboub-Schulte S, Thomas J. Weight-related teasing and internalized weight stigma predict abnormal eating attitudes and behaviours in Emirati female university students. Appetite 2016;102:44-50.

41. Vartanian LR, Porter AM. Weight stigma and eating behavior: A review of the literature. Appetite 2016;102:3-14.

42. Durso LE, Latner JD, Hayashi K. Perceived discrimination is associated with binge eating in a community sample of non-overweight, overweight, and obese adults. Obes Facts 2012;5(6):869-80.

43. Johnson SM, Edwards KM, Gidycz CA. Interpersonal weight-related pressure and disordered eating in college women: a test of an expanded tripartite influence model. Sex Roles 2015;72:15-24.

44. Culbert KM, Racine SE, Klump KL. Research review: what we have learned about the causes of eating disorders – a synthesis of sociocultural, psychological, and biological research. J Child Psychol Psychiatry 2015;56:1141-64.

45. Hsu LKG. Can dieting cause an eating disorder? Psychol Med 1997;27:509-13.

46. Stice E, Burger K. Dieting as a risk factor for eating disorders. In: Smolak L, Levine M, editors. The Wiley handbook of eating disorders. 1st ed. West Sussex: John Wiley and Sons;

2015. p. 312-23.

47. Polivy J, Herman CP. Dieting and binging: a causal analysis. Am Psychol 1985;40:193-201.

48. Wardle J, Beales S. Control and loss of control over eating: An experimental investigation. J Abnorm Psychol 1988;97:35.

49. Neumark-Sztainer D, Wall M, Guo J, Story M, Haines J, Eisenberg M. Obesity, disordered eating, and eating disorders in a longitudinal study of adolescents: how do dieters fare 5 years later? J Amer Diet Assoc 2006;106:559-68.

50. Liechty JM, Lee MJ. Longitudinal predictors of dieting and disordered eating among young adults in the U.S. Int J Eat Disord 2013;46:790-800.

51. O’Hara L, Taylor J. What’s wrong with the ‘War on Obesity?’: a narrative review of the weight-centered health paradigm and development of the 3C framework to build critical competency for a paradigm shift. SAGE Open 2018;8:2158244018772888.

52. Bacon L, Aphramor L. Weight science: evaluating the evidence for a paradigm shift. Nutr J 2011;10:9.

53. Mensinger JL, Calogero RM, Stranges S, Tylka TL. A weight-neutral versus weight-loss approach for health promotion in women with high BMI: A randomized-controlled trial. Appetite 2016;105:364-74.

54. Bacon L, Stern JS, Van Loan MD, Keim NL. Size acceptance and intuitive eating improve health for obese, female chronic dieters. J Amer Diet Assoc 2005;105:929-36.

55. Køster-Rasmussen R, Simonsen MK, Siersma V, Henriksen JE, Heitmann BL, Niels de Fine O. Intentional weight loss and longevity in overweight patients with Type 2 diabetes: a population-based cohort study. PLoS One 2016;11:e0146889.

56. Mann T, Tomiyama AJ, Westling E, Lew AM, Samuels B, Chatman J. Medicare's search for effective obesity treatments: diets are not the answer. Am Psychol 2007;62:220-33.

57. Garner DM, Wooley SC. Confronting the failure of behavioral and dietary treatments for obesity. Clin Psychol Rev 1991;11:729-80.

58. Tomiyama AJ, Ahlstrom B, Mann T. Long-term effects of dieting: is weight loss related to health? Soc Personal Psychol Compass 2013;7:861-77.

59. Wannamethee SG, Shaper AG, Walker M. Weight change, weight fluctuation, and mortality. Arch Intern Med 2002;162:2575-80.

60. Cologne J, Takahashi I, French B, Nanri A, Misumi M, Sadakane A, Cullings, HM, Araki, Y, Mizoue, T. Association of weight fluctuation with mortality in Japanese adults. JAMA Netw Open 2019;2(3):e190731-e.

61. Diaz VA, Mainous AG, Everett CJ. The association between weight fluctuation and mortality: results from a population-based cohort study. J Community Health. 2005;30:153-65.

62. Droyvold WB, Lund Nilsen TI, Lydersen S, Midthjell K, Nilsson PM, Nilsson JA, Holmen, J. Weight change and mortality: the Nord-Trondelag health study. J Intern Med 2005;257:338-45.

63. Andres R, Muller DC, Sorkin JD. Long-term effects of change in body weight on all-cause mortality: a review. Ann Intern Med 1993;119:737-43.

64. O'Dea JA. Prevention of child obesity: ‘First, do no harm’. Health Educ Res 2004;20:259-65.

65. Bonell C, Jamal F, Melendez-Torres GJ, Cummins S. 'Dark logic': theorising the harmful consequences of public health interventions. J Epidemiol Community Health. 2015;69:95-8.

66. Yager J, Devlin MJ, Halmi KA, Herzog DB, Mitchell JE, Powers P, Zerbe K. Guideline watch (August 2012): Practice guideline for the treatment of patients with eating disorders, 3rd Edition. FOCUS 2014;12:416-31.

67. Fairburn C. Eating Disorder Examination (Edition 16.0 D) and Eating Disorder Examination Questionnaire (EDE-Q 6.0) Cognitive therapy and eating disorders New York: Guilford Press; 2009. p. 265-313.

68. Berg KC, Peterson CB, Frazier P, Crow SJ. Psychometric evaluation of the eating disorder examination and eating disorder examination-questionnaire: a systematic review of the literature. Int J Eat Dis 2012;45:428-38.

69. Tregarthen J, Paik Kim J, Sadeh-Sharvit S, Neri E, Welch H, Lock J. Comparing a tailored self-help mobile app with a standard self-monitoring app for the treatment of eating disorder symptoms: randomized controlled trial. JMIR Ment Health 2019;6:e14972-e.

70. Hayes NA, Welty LJ, Slesinger N, Washburn JJ. Moderators of treatment outcomes in a partial hospitalization and intensive outpatient program for eating disorders. Eat Disord 2019;27(3):305-20.

71. Dahlgren CL, Stedal K, Rø Ø. Eating Disorder Examination Questionnaire (EDE-Q) and Clinical Impairment Assessment (CIA): clinical norms and functional impairment in male and female adults with eating disorders. Nord J Psychiatry 2017;71:256-61.

72. Braun V, Clarke V. Using thematic analysis in psychology. Qual Res Psychol 2006;3:77-101.

73. Singer JD, Willett JB. Applied longitudinal data analysis: modeling change and event occurrence: Oxford University Press; 2003.

74. Jacobi C, Hayward C, de Zwaan M, Kraemer HC, Agras WS. Coming to terms with risk factors for eating disorders: application of risk terminology and suggestions for a general taxonomy. Psychol Bull 2004;130:19-65.

75. Lie SØ, Rø Ø, Bang L. Is bullying and teasing associated with eating disorders? A systematic review and meta-analysis. Int J Eat Disord 2019;52:497-514.

76. Afifi TO, Sareen J, Fortier J, Taillieu T, Turner S, Cheung K, Henriksen CA. Child maltreatment and eating disorders among men and women in adulthood: results from a nationally representative United States sample. Int J Eat Disord 2017;50:1281-96.

77. Haynos AF, Wang SB, Lipson S, Peterson CB, Mitchell JE, Halmi KA, et al. Machine learning enhances prediction of illness course: a longitudinal study in eating disorders. Psychol Med 2020:1-11.

78. Sikorski C, Spahlholz J, Hartlev M, Riedel-Heller SG. Weight-based discrimination: an ubiquitary phenomenon? Int J Obes 2015; 40, 333-337.

79. Tylka TL, Annunziato RA, Burgard D, Danielsdottir S, Shuman E, Davis C, Calogero RM. The Weight-Inclusive versus Weight-Normative approach to health: evaluating the evidence for prioritizing well-being over weight loss. J Obes 2014;2014:983495.

80. Martin E, Dourish CT, Rotshtein P, Spetter MS, Higgs S. Interoception and disordered eating: A systematic review. Neurosci Biobehav Rev 2019;107:166-91.

81. Mensinger JL, Calogero RM, Tylka TL. Internalized weight stigma moderates eating behavior outcomes in women with high BMI participating in a healthy living program. Appetite 2016;102:32-43.

82. Puhl RM, Neumark-Sztainer D, Austin SB, Luedicke J, King KM. Setting policy priorities to address eating disorders and weight stigma: views from the field of eating disorders and the US general public. BMC Public Health 2014;14.

83. Austin SB. The blind spot in the drive for childhood obesity prevention: bringing eating disorders prevention into focus as a public health priority. Am J Public Health 2011;101:e1-4.

84. Alberga AS, Pickering BJ, Alix Hayden K, Ball GD, Edwards A, Jelinski S, Nutter S, Oddie S, Sharma AM, Russel-Mayhew SN. Weight bias reduction in health professionals: a systematic review. Clin Obes 2016;6:175-88.

85. Calogero RM, Tylka TL, Mensinger JL, Meadows A, Daníelsdóttir S. Recognizing the fundamental right to be fat: A weight-inclusive approach to size acceptance and healing from sizeism. Women Ther 2019;42:22-44.

86. Anderson LK, Reilly EE, Berner L, Wierenga CE, Jones MD, Brown TA, Kaye W, Cusack A. Treating eating disorders at higher levels of care: overview and challenges. Curr Psychiatry Rep 2017;19:48.

87. Mensinger JL, Tylka TL, Calamari ME. Mechanisms underlying weight status and healthcare avoidance in women: a study of weight stigma, body-related shame and guilt, and healthcare stress. Body Image 2018;25:139-47.

88. Bombak A. Obesity, health at every size, and public health policy. Am J Public Health 2014;104(2):e60-7.

89. Mann T, Tomiyama AJ, Ward A. Promoting public health in the context of the "Obesity Epidemic": false starts and promising new directions. Perspect Psychol Sci 2015;10:706-10.

FIGURE CAPTIONS

Figure 1. STROBE – Strengthening the Reporting of Observational Studies in Epidemiology; RTC – Residential Treatment Center; PHP – Partial Hospital Program; IOP – Intensive Outpatient Program


Note. STROBE – Strengthening the Reporting of Observational Studies in Epidemiology; RTC – Residential Treatment Center; PHP – Partial Hospital Program; IOP – Intensive Outpatient Program

Figure 2. Estimated Global EDE-Q Score Trajectories of Change.

Dashed line (No) – Patient not attributing eating disorder onset to anti-obesity messaging. Solid line (Yes) – Patient attributing eating disorder onset to anti-obesity messaging. Error bars represent 95% Confidence Intervals. First point represents predicted intake global EDE-Q (Eating Disorder Examination - Questionnaire) score for patient entering residential treatment; second point represents predicted residential discharge score after median length of stay (38 Days); third point represents predicted partial hospital discharge score after median length of stay (73 Days); fourth point represents predicted intensive outpatient program discharge score after median length of stay (119 Days).

*Color image is available online only.*
