# Supplementary material for: Treatment Outcomes and Trajectories of Change in Patients Attributing Their Eating Disorder Onset to Anti-obesity Messaging
Source: Psychosom Med. 2021 Jun 19;83(7):777–86. doi: 10.1097/PSY.0000000000000962 (PMC8428859; doi:10.1097/PSY.0000000000000962)
Supplement: SUPPLEMENTARY MATERIAL [file psymed-83-777-s002.docx]

| **Supplemental Digital Content 1** |  |  |  |  |  |  |
| --- | --- | --- | --- | --- | --- | --- |
| *Multilevel Models Predicting Global EDE-Q Scores Over Treatment* | | | | | | |
|  | **Model 1** | | **Model 2** | | **Model 3^☥^** | |
|  | **γ (SE)** | ***p*** | **γ (SE)** | ***p*** | **γ (SE)** | ***p*** |
| **Fixed Effects** |  |  |  |  |  | |
| **Model for Initial Status (EDE-Q score at admission)** | | | |  |  |  |
| **Intercept^†^** | 3.709 (0.038) | <.001 | 4.018 (0.075) | <.001 | 4.793 (0.115) | <.001 |
| Initial Level of Care - PHP | -0.365 (0.088) | <.001 | -0.345 (0.088) | <.001 | -0.386 (0.082) | <.001 |
| Initial Level of Care - IOP | -0.677 (0.108) | <.001 | -0.663 (0.108) | <.001 | -0.685 (0.105) | <.001 |
| Initial Level of Care - RTC (ref) | -- | -- | -- | -- | -- | -- |
| Anti-Obesity Messaging - No |  |  | -0.463 (0.086) | <.001 | -0.357 (0.080) | <.001 |
| Anti-Obesity Messaging - Unsure |  |  | -0.288 (0.089) | .001 | -0.223 (0.082) | .007 |
| Anti-Obesity Messaging - Yes (ref) |  |  | -- | -- | -- | -- |
| ***Covariates*** |  |  |  |  |  |  |
| Age 19 and Older |  |  |  |  | 0.255 (0.066) | <.001 |
| Age 18 and Younger (ref) |  |  |  |  | -- | -- |
| Male |  |  |  |  | -0.657 (0.128) | <.001 |
| Female (ref) |  |  |  |  | -- | -- |
| Latinx/Hispanic |  |  |  |  | -0.076 (0.093) | .41 |
| Asian/Pacific Islander |  |  |  |  | -0.272 (0.145) | .061 |
| African American/Black |  |  |  |  | -0.120 (0.232) | .61 |
| Multiracial/Biracial |  |  |  |  | 0.022 (0.166) | .90 |
| American Indian/Native American |  |  |  |  | -1.116 (0.398) | .005 |
| White Non-Hispanic (ref) |  |  |  |  | -- | -- |
| Anorexia Nervosa-Restrictive subtype |  |  |  |  | -0.639 (0.086) | <.001 |
| Bulimia Nervosa |  |  |  |  | -0.400 (0.102) | <.001 |
| Binge Eating Disorder |  |  |  |  | -1.006 (0.164) | <.001 |
| ARFID |  |  |  |  | -2.664 (0.193) | <.001 |
| OSFED |  |  |  |  | -0.732 (0.114) | <.001 |
| Anorexia Nervosa-Purging subtype (ref) |  |  |  |  | -- | -- |
| No Prior Eating Disorder Treatment |  |  |  |  | 0.158 (0.075) | .036 |
| Prior Eating Disorder Treatment (ref) |  |  |  |  | -- | -- |
| No History of Sexual Abuse |  |  |  |  | -0.273 (0.071) | <.001 |
| Sexual Abuse History (ref) |  |  |  |  | -- | -- |
| No History of Being Bullied |  |  |  |  | -0.203 (0.053) | <.001 |
| History of Being Bullied (ref) |  |  |  |  | -- | -- |
| Months Since Eating Disorder Onset^§^ |  |  |  |  | 0.059 (0.026) | .021 |
| Intake Percent Target Body Weight^§^ |  |  |  |  | 0.193 (0.037) | <.001 |
| **Model for Rate of Acceleration (slope during phase 1)** | | | |  |  |  |
| **Intercept^††^** | 1.364 (0.053) | <.001 | 1.368 (0.053) | <.001 | 1.979 (0.100) | <.001 |
| Initial Level of Care - PHP | -0.382 (0.112) | .001 | -0.392 (0.112) | <.001 | -0.372 (0.110) | .001 |
| Initial Level of Care - IOP | -0.551 (0.228) | .016 | -0.549 (0.228) | .016 | -0.528 (0.224) | .019 |
| Initial Level of Care - RTC (ref) | -- | -- | -- | -- | -- | -- |
| ***Covariates*** |  |  |  |  |  |  |
| Male |  |  |  |  | -0.276 (0.133) | .037 |
| Female (ref) |  |  |  |  | -- | -- |
| Latinx/Hispanic |  |  |  |  | -0.069 (0.133) | .601 |
| Asian/Pacific Islander |  |  |  |  | -0.068 (0.217) | .76 |
| African American/Black |  |  |  |  | -0.733 (0.365) | .045 |
| Multiracial/Biracial |  |  |  |  | -0.720 (0.231) | .002 |
| American Indian/Native American |  |  |  |  | -0.238 (0.527) | .65 |
| White Non-Hispanic (ref) |  |  |  |  | -- | -- |
| Anorexia Nervosa-Restrictive subtype |  |  |  |  | -0.542 (0.088) | <.001 |
| *continued on next page* |  |  |  |  |  |  |

| **Supplemental Digital Content 1, cont.** |  |  |  |  |  |  |
| --- | --- | --- | --- | --- | --- | --- |
| *Multilevel Models Predicting Global EDE-Q Scores Over Treatment* | | | | | | |
|  | **Model 1** | | **Model 2** | | **Model 3** | |
| Bulimia Nervosa |  |  |  |  | -0.065 (0.102) | .53 |
| Binge Eating Disorder |  |  |  |  | -0.294 (0.144) | .042 |
| ARFID |  |  |  |  | -1.175 (0.196) | <.001 |
| OSFED |  |  |  |  | -0.447 (0.145) | <.001 |
| Anorexia Nervosa-Purging subtype (ref) |  |  |  |  | -- | -- |
| No Prior Eating Disorder Treatment |  |  |  |  | 0.287 (0.109) | .008 |
| Prior Eating Disorder Treatment (ref) |  |  |  |  | -- | -- |
| No History of Sexual Abuse |  |  |  |  | -0.264 (0.072) | <.001 |
| History of Sexual Abuse (ref) |  |  |  |  | -- | -- |
| No History of Other Trauma |  |  |  |  | -0.161 (0.058) | .005 |
| History of Other Trauma (ref) |  |  |  |  | -- | -- |
| **Model for Rate of Change (slope during phase 2)** | | | |  |  |  |
| **Intercept^*^** | -0.004 (0.001) | <.001 | -0.007 (0.001) | <.001 | -0.006 (0.001) | <.001 |
| Initial Level of Care - PHP | -0.005 (0.001) | .001 | -0.005 (0.001) | <.001 | -0.005 (0.001) | .001 |
| Initial Level of Care - IOP | -0.004 (0.003) | .203 | -0.004 (0.003) | .194 | -0.003 (0.003) | .39 |
| Initial Level of Care - RTC (ref) | -- | -- | -- | -- | -- | -- |
| Anti-Obesity Messaging - No |  |  | 0.003 (0.001) | .008 | 0.003 (0.001) | .016 |
| Anti-Obesity Messaging - Unsure |  |  | 0.003 (0.001) | .014 | 0.003 (0.001) | .015 |
| Anti-Obesity Messaging -Yes (ref) |  |  | -- | -- | -- | -- |
| ***Covariates*** |  |  |  |  |  |  |
| Latinx/Hispanic |  |  |  |  | 0.000 (0.002) | .87 |
| Asian/Pacific Islander |  |  |  |  | 0.003 (0.003) | .27 |
| African American/Black |  |  |  |  | -0.009 (0.006) | .13 |
| Multiracial/Biracial |  |  |  |  | -0.009 (0.003) | .007 |
| American Indian/Native American |  |  |  |  | 0.015 (0.007) | .041 |
| White Non-Hispanic (ref) |  |  |  |  | -- | -- |
| No Prior Eating Disorder Treatment |  |  |  |  | -0.004 (0.002) | .008 |
| Prior Eating Disorder Treatment (ref) |  |  |  |  | -- | -- |
| **Random Effects** |  |  |  |  |  |  |
| **Level 1** | **σ^2^ (SE)** | ***p*** | **σ^2^ (SE)** | ***p*** | **σ^2^ (SE)** | ***p*** |
| Within-person | 0.215 (0.012) | <.001 | 0.215 (0.012) | <.001 | 0.217 (0.013) | <.001 |
| **Level 2** | **τ (SE)** | ***p*** | **τ (SE)** | ***p*** | **τ (SE)** | ***p*** |
| Initial status | 2.454 (0.077) | <.001 | 2.425 (0.076) | <.001 | 1.971 (0.064) | <.001 |
| Rate of acceleration (slope 1) | 2.241 (0.131) | <.001 | 2.244 (0.131) | <.001 | 2.079 (0.126) | <.001 |
| Rate of change (slope 2) | 0.0002 (0.000) | <.001 | 0.0002 (0.000) | <.001 | 0.0002 (0.000) | <.001 |
| **Goodness-of-Fit Statistics** |  |  |  |  |  |  |
| -2 Log Likelihood | 19822.1 |  | 19790.9 |  | 19214.1 |  |
| Akaike's Information Criterion | 19854.1 |  | 19830.9 |  | 19328.1 |  |
| *Note.* After adjustments for covariate missingness, N=2761; Model 1 represents the unconditional growth model (with entry level-of-care controlled to account for sample structure), Model 2 tests Aim 3a, 3b, and 3c, Model 3 is confounder-adjusted. ^†^Interpretation of Intercept for Initial Status Model is the mean admission EDE-Q score for the patient who entered at the residential level-of-care. ^††^Interpretation of Intercept for Rate of Acceleration Model is the average initial improvement for the patient entering the residential level-of-care. ^*^Interpretation of Intercept for Rate of Change Model is the average improvement by discharge from final level-of-care for the patient entering residential treatment. ^§^Standardized variables. ^☥^Model 3 intercepts represent an individual who scores at the mean on the continuous variables and falls into the reference group on the categorical variables. EDE-Q – Eating Disorder Examination-Questionnaire; PHP – Partial Hospital Program; IOP – Intensive Outpatient Program; RTC – Residential Treatment Center; ref – Reference group; ARFID – Avoidant Restrictive Food Intake Disorder; OSFED – Other Specified Feeding and Eating Disorder. | | | | | | |
