# Supplementary material for: Treatment Outcomes and Trajectories of Change in Patients Attributing Their Eating Disorder Onset to Anti-obesity Messaging
Source: Psychosom Med. 2021 Jun 19;83(7):777–86. doi: 10.1097/PSY.0000000000000962 (PMC8428859; doi:10.1097/PSY.0000000000000962)
Supplement: SUPPLEMENTARY MATERIAL [file psymed-83-777-s003.docx]

| **Supplemental Digital Content 2** | | | | | | | | | | |
| --- | --- | --- | --- | --- | --- | --- | --- | --- | --- | --- |
| *Confounder-Adjusted Estimated Marginal Mean Global EDE-Q Scores* | | | | | | | | | | |
|  | | **Anorexia Nervosa – Restricting subtype** | | | | | | | | |
|  | |  | Yes (n=219/18%) | |  | No (n=572) | |  | Unsure (n=449) | |
| Treatment Stage | Day | | EM Mean | 95% CI |  | EM Mean | 95% CI |  | EM Mean | 95% CI |
| RTC Admission | 0 | | 3.49 | (3.23, 3.75) |  | 3.13 | (2.89, 3.36) |  | 3.27 | (3.03, 3.51) |
| RTC Discharge | 38 | | 2.30 | (2.03, 2.57) |  | 2.07 | (1.83, 2.31) |  | 2.18 | (1.94, 2.43) |
| PHP Discharge | 73 | | 2.03 | (1.75, 2.31) |  | 1.89 | (1.64, 2.14) |  | 2.03 | (1.78, 2.29) |
| IOP Discharge | 119 | | 1.68 | (1.30, 2.07) |  | 1.66 | (1.31, 2.02) |  | 1.85 | (1.48, 2.22) |
|  | | **Anorexia Nervosa – Binge/Purge subtype** | | | | | | | | |
|  | |  | Yes (n=87/18%) | |  | No (n=237) | |  | Unsure (n=171) | |
| Treatment Stage | Day | | EM Mean | 95% CI |  | EM Mean | 95% CI |  | EM Mean | 95% CI |
| RTC Admission | 0 | | 4.13 | (3.85, 4.40) |  | 3.77 | (3.51, 4.02) |  | 3.91 | (3.66, 4.17) |
| RTC Discharge | 38 | | 2.41 | (2.13, 2.70) |  | 2.18 | (1.92, 2.44) |  | 2.30 | (2.03, 2.56) |
| PHP Discharge | 73 | | 2.14 | (1.85, 2.43) |  | 1.99 | (1.73, 2.63) |  | 2.14 | (1.86, 2.42) |
| IOP Discharge | 119 | | 1.79 | (1.39, 2.18) |  | 1.76 | (1.40, 2.13) |  | 1.95 | (1.56, 2.33) |
|  | | **Bulimia Nervosa** | | | | | | | | |
|  | |  | Yes (n=101/19%) | |  | No (n=220) | |  | Unsure (n=211) | |
| Treatment Stage | Day | | EM Mean | 95% CI |  | EM Mean | 95% CI |  | EM Mean | 95% CI |
| RTC Admission | 0 | | 3.73 | (3.46, 4.00) |  | 3.37 | (3.12, 3.62) |  | 3.51 | (3.26, 3.77) |
| RTC Discharge | 38 | | 2.08 | (1.80, 2.36) |  | 1.85 | (1.59, 2.11) |  | 1.96 | (1.70, 2.22) |
| PHP Discharge | 73 | | 1.80 | (1.51, 2.10) |  | 1.66 | (1.39, 1.93) |  | 1.80 | (1.53, 2.08) |
| IOP Discharge | 119 | | 1.45 | (1.05, 1.85) |  | 1.43 | (1.06, 1.80) |  | 1.61 | (1.23, 2.00) |
|  | | **Binge Eating Disorder** | | | | | | | | |
|  | |  | Yes (n=34/17%) | |  | No (n=101) | |  | Unsure (n=62) | |
| Treatment Stage | Day | | EM Mean | 95% CI |  | EM Mean | 95% CI |  | EM Mean | 95% CI |
| RTC Admission | 0 | | 3.12 | (2.76, 3.49) |  | 2.76 | (2.42, 3.10) |  | 2.91 | (2.56, 3.26) |
| RTC Discharge | 38 | | 1.69 | (1.32, 2.06) |  | 1.46 | (1.11, 1.81) |  | 1.57 | (1.22, 1.93) |
| PHP Discharge | 73 | | 1.42 | (1.04, 1.80) |  | 1.28 | (0.92, 1.63) |  | 1.42 | (1.05, 1.79) |
| IOP Discharge | 119 | | 1.07 | (0.60, 1.54) |  | 1.05 | (0.61, 1.48) |  | 1.23 | (0.78, 1.68) |
|  | | **Other Specified Feeding and Eating Disorder** | | | | | | | | |
|  | |  | Yes (n=73/22%) | |  | No (n=133) | |  | Unsure (n=130) | |
| Treatment Stage | Day | | EM Mean | 95% CI |  | EM Mean | 95% CI |  | EM Mean | 95% CI |
| RTC Admission | 0 | | 3.40 | (3.11, 3.69) |  | 3.04 | (2.76, 3.31) |  | 3.18 | (2.91, 3.45) |
| RTC Discharge | 38 | | 2.12 | (1.82, 2.42) |  | 1.89 | (1.61, 2.16) |  | 2.00 | (1.72, 2.28) |
| PHP Discharge | 73 | | 1.85 | (1.54, 2.15) |  | 1.71 | (1.42, 1.99) |  | 1.85 | (1.56, 2.14) |
| IOP Discharge | 119 | | 1.50 | (1.09, 1.91) |  | 1.48 | (1.10, 1.86) |  | 1.66 | (1.27, 2.06) |
| *continued on next page* | | | | | | | | | | |
| **Supplemental Digital Content 2, cont.**  *Confounder-Adjusted Estimated Marginal Mean Global EDE-Q Scores* | | | | | | | | | | |
|  | | **Avoidant Restrictive Food Intake Disorder** | | | | | | | | |
|  | |  | Yes (n=7/8%) | |  | No (n=47) | |  | Unsure (n=29) | |
| Treatment Stage | Day | | EM Mean | 95% CI |  | EM Mean | 95% CI |  | EM Mean | 95% CI |
| RTC Admission | 0 | | 1.47 | (1.05, 1.88) |  | 1.10 | (0.71, 1.5) |  | 1.25 | (0.85, 1.65) |
| RTC Discharge | 38 | | 0.89 | (0.48, 1.31) |  | 0.66 | (0.26, 1.06) |  | 0.78 | (0.37, 1.18) |
| PHP Discharge | 73 | | 0.63 | (0.21, 1.05) |  | 0.49 | (0.08, 0.89) |  | 0.63 | (0.22, 1.04) |
| IOP Discharge | 119 | | 0.29 | (-0.22, 0.79) |  | 0.26 | (-0.21, 0.74) |  | 0.45 | (-0.04, 0.94) |

*Note*. Days represent median discharge times for patients enrolled in all three levels-of-care. EM – Estimated Marginal; EDE-Q – Eating Disorder Examination Questionnaire; RTC – Residential Treatment Center; PHP – Partial Hospital Program; IOP – Intensive Outpatient Program.

Percentage value after the sample size in the Yes column shows the proportion of the patients with that diagnosis who attributed their ED trigger to anti-obesity messages. Covariates in the model included age, gender identity, race, months since ED onset, prior ED treatment, % target body weight at intake, trauma history, sexual abuse history, and bullying history
