# Supplementary material for: Treatment Outcomes and Trajectories of Change in Patients Attributing Their Eating Disorder Onset to Anti-obesity Messaging
Source: Psychosom Med. 2021 Jun 19;83(7):777–86. doi: 10.1097/PSY.0000000000000962 (PMC8428859; doi:10.1097/PSY.0000000000000962)
Supplement: SUPPLEMENTARY MATERIAL [file psymed-83-777-s004.docx]

**Supplemental Digital Content 3**

*Confounder-Adjusted* *Estimated* *Global EDE-Q Score Trajectories of Change*

#
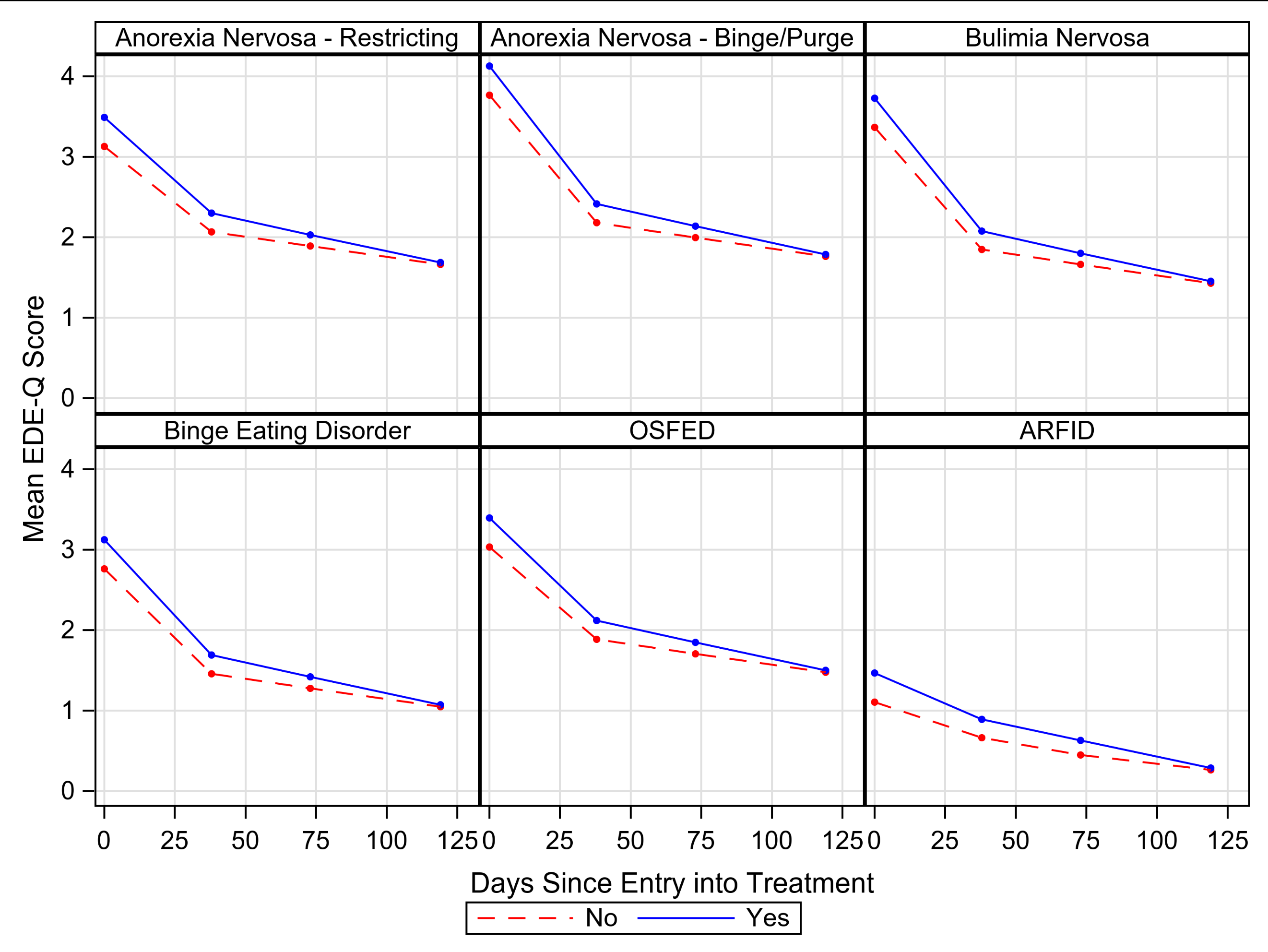


*Note.* Dashed line (No) – Patient not attributing eating disorder onset to anti-obesity messaging. Solid line (Yes) – Patient attributing eating disorder onset to anti-obesity messaging. First point represents estimated intake EDE-Q (Eating Disorder Examination - Questionnaire) score for patient entering residential treatment; second point represents estimated residential discharge score after median length of stay (38 Days); third point represents estimated partial hospital discharge score after median length of stay (73 Days); fourth point represents discharge intensive outpatient program discharge score after median length of stay (119 Days). Covariates in the model included age, gender identity, race, months since ED onset, prior ED treatment, % target body weight at intake, trauma history, sexual abuse history, and bullying history.
